# Photosensitizers Mediated Photodynamic Inactivation against Fungi

**DOI:** 10.3390/nano11112883

**Published:** 2021-10-28

**Authors:** Daniel Ziental, Dariusz T. Mlynarczyk, Beata Czarczynska-Goslinska, Konrad Lewandowski, Lukasz Sobotta

**Affiliations:** 1 Chair and Department of Inorganic and Analytical Chemistry, Poznan University of Medical Sciences, Grunwaldzka 6, 60-780 Poznan, Poland; dziental@op.pl (D.Z.); konradlewandowski.lew@gmail.com (K.L.); 2 Chair and Department of Chemical Technology of Drugs, Poznan University of Medical Sciences, Grunwaldzka 6, 60-780 Poznan, Poland; mlynarczykd@ump.edu.pl; 3 Chair and Department of Pharmaceutical Technology, Poznan University of Medical Sciences, Grunwaldzka 6, 60-780 Poznan, Poland; bgoslinska@ump.edu.pl

**Keywords:** macrocycle, photocytotoxicity, fungi, photodynamic therapy, infections

## Abstract

Superficial and systemic fungal infections are essential problems for the modern health care system. One of the challenges is the growing resistance of fungi to classic antifungals and the constantly increasing cost of therapy. These factors force the scientific world to intensify the search for alternative and more effective methods of treatment. This paper presents an overview of new fungal inactivation methods using Photodynamic Antimicrobial Chemotherapy (PACT). The results of research on compounds from the groups of phenothiazines, xanthanes, porphyrins, chlorins, porphyrazines, and phthalocyanines are presented. An intensive search for a photosensitizer with excellent properties is currently underway. The formulation based on the existing ones is also developed by combining them with nanoparticles and common antifungal therapy. Numerous studies indicate that fungi do not form any specific defense mechanism against PACT, which deems it a promising therapeutic alternative.

## 1. Introduction

Recently, it has been reported that more than 300 million people all over the world suffer from severe fungal infections. Simultaneously, 1.6 million deaths worldwide are related to fungal infections each year. It is estimated that ca. 1.7 billion people undergo superficial fungal infections (skin, mucosa, hair, nail). Certain health conditions produce increased susceptibility to fungal infections, including immune system suppression, i.e., progress of HIV/AIDS, hematologic cancers, and the period after transplantations. The second important predisposing factor is the dysbiosis of natural microbiome which among others may be caused by the exposure to antimicrobials. Fungal infections are also associated with the use of medical devices such as stents or catheters [1,2,3,4]. Superficial fungal infections can cause life-threatening problems [5]. Oral candidiasis alone is becoming increasingly common with patient age. *Candida albicans*, although naturally occurring in the oral microbiota of 60–70% of the population, may in some conditions overgrow and cause health problems to the host [6,7]. *Candida* spp. are responsible for most of the invasive fungal infections, including life-threatening candidemia. It is known that *Candida* spp. easily form hard to treat biofilm [3]. Another growing issue is the occurrence of infections of keratinized tissues (skin, nail) caused by dermatophytes, whose incidence is on the rise and thus are becoming a serious problem in the society [8].

Currently, four classes of antifungal drugs are used in clinical practice. These are: polyenes, which disrupt ergosterol in the fungal cell membranes; azoles, which inhibit lanosterol 14α-demethylase (enzyme converting lanosterol to ergosterol); pyrimidine analogs, which convert cytosine deaminase to 5-fluorouracil (antimetabolite hampering RNA and DNA synthesis); echinocandins, which inhibit β-(1,3)-D-glucan synthase. Unfortunately, treatment with antifungal drugs often must continue for a prolonged period of time to prevent the recurrence. Because of that, toxic side effects are expected along with resistance development by fungal cells. The presence of efflux pump has also been reported [1]. The abovementioned biofilm is also a significant factor contributing to fungal drug resistance. Biofilm structure appears when cells stick to each other and extracellular polymeric substances (EPS) are produced, which act as a binder and keep the cells aggregate together. Moreover, EPS is considered a barrier hampering drug distribution within the biofilm structure. Because of that, biofilm is more resistant to treatment in comparison with a planktonic form of fungi. Additionally, cells in biofilm more efficiently resist the host immune system. To achieve such high level of self-defense, biofilm cells have to communicate with each other. To visualize the character of this structure, Watnik and Kolter termed biofilm a “city of microbes” [3,6,9].

In the light of abovementioned facts, scientists are searching for new drugs against fungi. The more traditional approach is to modify known drugs to achieve more active molecules. However, fungi—just as mammalian cells—are Eukaryotes, as their cells have a nucleus coated by a nuclear envelope. Structural similarities between fungal and human cells lead to difficulties in fungal infections treatment [5]. Therefore, new treatment protocols with antifungal drugs combinations are researched and introduced. It is considered to utilize immune cells as a cell-based therapy as well as cytokines as an adjuvant tool [1]. Researchers are developing molecules to fight with fungi by destroying new targets such as inositol phosphatyl-ceramide synthase, inositol acyltransferase, acetohydroxyacid synthase, and many others [2].

One of the new promising antifungal treatments is photodynamic antimicrobial chemotherapy (PACT) [9]. PACT is based on the same principles as photodynamic therapy (PDT) and relies on the interaction between the photosensitizer (PS), molecular oxygen and light, which leads to reactive oxygen species (ROS) formation. ROS are responsible for disrupting/destroying life-essential cell structures [3,10,11]. PACT thanks to its mechanism of action via singlet oxygen reveals multi-target activity against fungi. Moreover, fungal cells due to high exposition to singlet oxygen in relatively short time cannot develop resistance. Resistance in the microbial world is a key problem for present and future medicine. Importantly, PACT causes only insignificant side-effects. It should be highlighted that no genotoxic and mutagenic effects in human cells were found for the antifungal PACT conditions [4].

## 2. Mechanism of Photosensitizer Action against Fungi

**Figure 1:**
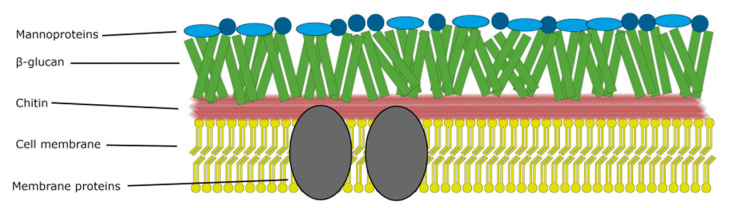
Structure of fungi cell wall.

After the light quant absorption, the PS molecule is transformed from its ground singlet state to the excited singlet state. The molecule with an excess of energy can release it in a few ways, both non-radiative and radiative. The latter may be executed by emitting fluorescence. As for the non-radiative, the energy may be dissipated by vibration and heat emission. The energy can be also transferred to other molecules, e.g., oxygen or nitrogen derivatives resulting in oxygen and nitrogen radicals formation (type I photodynamic reaction). Short-living excited singlet state of PS can also undergo intersystem crossing to yield excited triplet state PS, a long living form (meta-stable state) of a lower energy. Triplet state excited PS is capable of emitting phosphorescence or, just as before, energy transfer to other molecules. This enables it to transform molecular oxygen to singlet oxygen, which is a very reactive key agent in photodynamic methods responsible for killing cells (type II photodynamic reaction). The process of ROS formation has been widely described [12,13,14]. The great advantage of PACT is the fact that the PS upon activation produces ROS, which expresses multi-target action. Significantly, singlet oxygen (the key ROS molecule) is formed only under PS irradiation, its lifetime is ca. 10^−6^ s, and it can migrate up to 100 nm. The mentioned features prevent fungi to develop resistance most probably due to the very short exposition to ROS and the non-toxicity of the PS to fungal cells [3,4,9,15]. According to the presented basis of photodynamic process, the PACT requires the use of light of an appropriate wavelength. The light doses vary in the range from 10 to 200 J/cm^2^ or are even higher in some cases. To achieve such doses, the power output of the light source should be adjusted even up to 100 mW/cm^2^, which might induce local temperature increase resulting in pain or burn. Therefore, the use of antifungal PACT is limited to the nail, hair, skin and in some cases oral cavity [4]. In consequence, the impact area of singlet oxygen is limited; therefore, the need for efficient PS delivery systems rises. The most important PS carriers are liposomes briefly described by Skupin-Mrugalska et al. [16]. The rate of antifungal effect depends on log*P*, ionization degree, as well as the charge of the PS molecule [9]. Moreover, hydrophilic PSs bearing charges in the peripheral groups penetrate the fungal cell wall more easily [4]. To provide high effectiveness of PACT against fungi, higher concentrations of PS are required in comparison to antibacterial and antiviral PACT [17,18,19], which may be attributed to a few factors. First of all, structural differences may be taken into account: the fungal cell is up to 50 times larger than the bacterial one [4,9], and the wall structure differs in bacteria and fungi. The wall of fungi cell consists of mannoproteins, *β*-glucan, chitin, and cell membrane (Figure 1). The chitin is a polymer of *N*-acetylglucosamine with high mechanical strength that provides inflexibility and compact nature to the fungal cell wall [20].

Due to differences in PS structure, certain PS groups target specific cell components in the fungal cells. Phenothiazine dyes affect mostly the cell membrane by doing damage to its components. On the other hand, porphyrins alter the membrane which enables them to enter the cell and achieve internal targets [5,21,22]. It was reported that neutral amphiphilic porphyrins and water soluble porphyrins bearing amino acids in the periphery were more prone to end up internalized into the cell more efficiently in comparison to the hydrophobic compounds, e.g., benzoporphyrins [9]. After crossing the cell wall, PS is located next to crucial inner structures, i.e., mitochondria, lysosomes, or nucleus. Subsequent activation with appropriate light length and light dose results in the destruction of the mentioned organelles leading to cell death [23]. However, in the case of fungi, the possibility of DNA damage is limited because of the presence of an additional membrane surrounding the nucleus, as the barrier hampers the dye penetration to the DNA [4,9]. Nevertheless, there are some reports about yeast DNA alterations occurring during PACT treatment [24]. Phthalocyanines have analogical site of action. As for the curcumin and its derivatives, it was observed that photodynamic process triggered with high light doses induced fungal cell death via necrosis, while the use of low light doses was associated with apoptosis [5,25]. Similar relationship for cancer cells was reported [26,27]. Photodynamic treatment causes a reduction in the secretion of yeast enzymes which impacts their virulence. It was noticed that PACT with methylene blue as a PS reduced the level of extracellular enzymes produced by *C. albicans*, and the concentration of proteinase was significantly decreased, whereas phospholipase formation was slightly lowered [28]. Both enzymes facilitate infections through increase of the adhesion to mammalian cells, interaction with human immune system, and disintegration of host cell membranes [29]. In another study, the photodynamic procedure with toluidine blue O as PS resulted in inactivation of alcohol dehydrogenase and cytochrome c oxidase, as well as in the decrease in ATP formation. Photodynamic treatment caused perturbations both in glycolysis and in fermentative pathways, which showed that PACT affects the cellular metabolic processes [30].

One of the biochemical bases of PACT’s effectiveness in fighting bacteria and fungi is the activation of the host immune system. The effect of PDT on the immune response in cancer treatment is beyond doubt and is well documented in the literature [31]. In photodynamic anticancer therapy, both pathways of the immune response are activated-innate and acquired immunity. In the case of bacterial and fungal infections, there is less research documenting the immunological aspect of PACT activity. Huang et al., in 2012, identified an important problem regarding the role of the immune response in PACT [32]. Commercial intravenous photosensitizers, including Photofrin, have a greater affinity for host tissues than for bacterial cells. Moreover, neutrophil cells show a strong affinity for some PS [32]. At the same time, neutrophils play a key role in fighting infections. Due to this relationship, the use of Photofrin-like photosensitizers may paradoxically weaken the host’s ability to combat bacteria and fungi. The issue discussed emphasizes the need to search for modern photosensitizers that could be commercialized and used in PACT.

In one of the in vivo studies, it was paradoxically observed that the use of methylene blue did not cause direct bacterial death but mobilized antibodies against neutrophils. Moreover, in the murine model of arthritis caused by *Staphylococcus aureus* (MRSA), the use of PACT and preventive PDT resulted in the accumulation of neutrophils that were actively involved in the fight against bacteria [32]. It is believed that implementation of PACT leads to the stimulation of the immune response against microbes [33]. *P. aeruginosa* infected wounds in mice were examined for markers of inflammation. It has been observed that as a result of the use of PACT, the expression of TLR-4, NF-kB, and proinflammatory interleukins decreases, while the level of FGF-2 and ALP increases [34]. In the case of PACT against fungal infections, the literature on the immune response is very limited. Some of the most interesting data come from studies on *G. mellonella* larvae. It was observed that larvae infected with *C. albicans* and then subjected to PACT show an increased presence of hemocytes [35]. These outcomes corresponded to an increased survival rate of the larvae. Similar results were found when the larvae were infected with microconidia *Fusarium keratoplasticum* and *Fusarium moniliforme* [36]. Regardless of the photosensitizers used, significant growth in hemolymph density and hemocytes concentration was observed.

**Figure 2:**
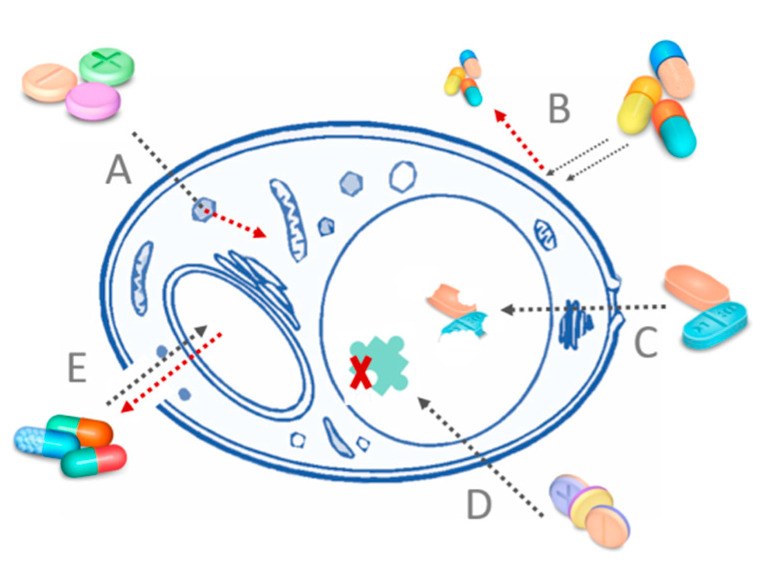
Mechanisms of fungal resistance: (**A**) The development of new mechanisms by fungi, which are no longer a target for antifungals. (**B**) Limiting the penetration of substances from the outside of the matrix. (**C**) Breakdown of antifungals by enzymes. (**D**) Changing the molecular target. (**E**) Pumping out molecules from the inside of the cell.

Fungi have developed resistance to some PSs similarly to bacteria (Figure 2). In cases of both microorganism types, the negative influence of multidrug efflux pumps was observed. The PS is removed from the cell, and the ROS are formed outside the cell thus greatly reducing the damage or even doing no damage to the cell. Thus far, it has been proposed to combine the PS with a compound known to inhibit the efflux pumps, i.e., verapamil. Moreover, extensive formation of enzymes deactivating oxygen radicals by fungal cells was noticed, but no mechanism of deactivating singlet oxygen in fungi has been reported yet [19,23].

This is one of the reasons that singlet oxygen is regarded as the main ROS thought to be responsible for effective PACT [4,37]. It is due to the fact that although many other ROS may be formed in the type I reaction by the PS (i.e., ^•^OH, O_2_^−•^, or H_2_O_2_), fungal cells have developed a series of both enzymatic and non-enzymatic antioxidant response mechanisms [38,39,40]. This is connected with the utilization of certain ROS by fungal cells in cell signaling for triggering a specific cell response, such as germination, development, or intercellular communication, but fungi are exposed to ROS originating from environmental, chemical, and physical stress factors [40]. Furthermore, certain strains of fungi were found to express increased production of hydroxyl radical as a form of defense from bacteria. The mechanisms that ensure proper regulation and prevent excessive oxidative stress include superoxide dismutases, catalases, glutathione peroxidases and reductases, thiol peroxidases, thio-, gluta-, sulfi- and peroxiredoxins, and production of secondary metabolites acting as radical scavengers [38,39,40]. However, this plethora of defense systems usually require prolonged exposure to be fully up-regulated and thus may be insufficient in case of a sudden burst of each of the ROS as an effect of PACT [41].

The damage induced by PACT are strongly related to the localization of the PS in the fungal cell and are dependent on multiple factors. It was established that PACT may lead to cell death due to the loss of functions of proteins and enzymes as a result of their oxidation and denaturation, peroxidation of lipids, which prevents proper functioning of mitochondria, lysosomes, or the lysis of cell membranes [42]. It was also observed that PACT may also induce cytotoxic, mutagenic, and carcinogenic effects in fungi, as well as change the expression of genes not directly associated with oxidative stress [41]. Unfortunately, detailed mechanisms are yet to be discovered.

## 3. Phenothiazine Photosensitizers

**Figure 3:**
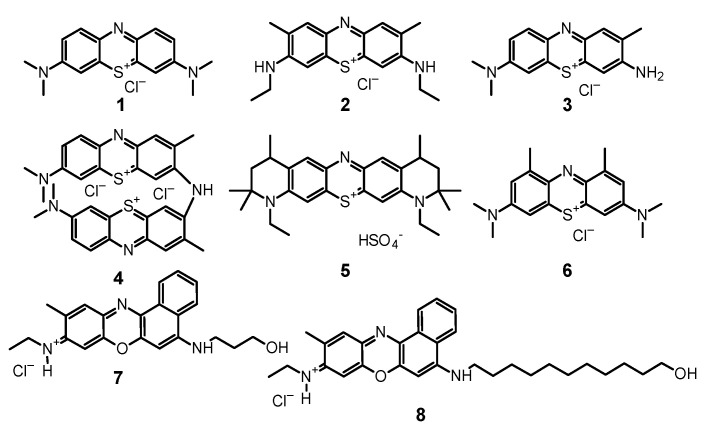
Chemical structures of (**1**–**8**).

Phenothiazines are among the oldest and best-known PSs that are successfully used in PDT. These compounds meet the key requirements needed in PACT, as they are stable in solutions, have an affinity for microbial cells, absorb light in the visible light range, and efficiently generate ROS [18]. Phenothiazines mostly absorb red light, which prevents the phenomenon of endogenous light absorption by body tissues in the infected area of the human body [43,44]. The maximum absorption of the most popular PS from the phenothiazine group—methylene blue (1, Figure 3)—is 660 nm, while toluidine blue is 625 nm [43]. The development of PSs focused, *inter alia*, on obtaining chemical derivatives with the absorption maximum shifted to further regions of the light spectrum. One of the significant successes was the production of new methylene blue (2, Figure 3) with maximum absorption over 800 nm. The achieved bathochromic shift not only reduces the abovementioned problem of endogenous absorption but also increases the usefulness of the PS in PACT. With the shift in the wavelength range, the light transmittance through the tissues increases and reaches the maximum value for light in the range from 800 to 900 nm.

### 3.1. Methylene Blue

**1** is a leading representative of the group of phenothiazine dyes, due to its long history of use in PDT, treatment of cyanide and carbon monoxide poisoning, and treatment of methemoglobinemia. An additional advantage of this compound is its outstanding singlet oxygen generation efficiency. Unfortunately, great results in the production of ROS in physicochemical tests do not always translate easily into PACT effectiveness. It should be strongly emphasized that methylene blue is not an ideal PS in PACT. A significant problem is its tendency to aggregate in solutions due to the planar chemical structure [43]. Currently, there are over 50 studies that focus on the fungicidal properties of **1**. In the last ten years, over 30 fungal species were assessed for their susceptibility to **1**-mediated PACT, many of which were found sensitive (Table 1).

The most studied fungal species in this manner has become *Candida albicans*. Sensitivity of these microorganisms to PACT has been established many times under different conditions by using **1** and the obtained results give a broad overview. *C. albicans* is a popular research subject because it is a useful reference for other species, and it is still one of the most common causes of candidosis and candidemia [35]. In recent experiments, not only the effectiveness of **1** as a fungicide was assessed but also its influence on the metabolic processes of *C. albicans* cells [45]. In the study by Freire et al., microorganisms isolated from a patient infected with HIV and a patient treated for denture stomatitis lesions, along with a reference strain, were subjected to PDT. The expression of several genes was investigated in the fungus: TEC1 (transcription factor), HWP1 (cell wall protein hyphae), EFG1 (transcriptional regulator related to morphogenesis), BCR1 (regulator of biofilm formation and cell wall), CPH1 (transcriptional regulator involved in morphogenesis), and ALS3 (adhesin). These genes are important determinants of the microorganism virulence, and a change in their expression helps to better understand how PACT impacts fungi. After applying **1** at the concentration of 300 μM and erythrosine (**9**) at 400 μM, the microorganism suspension was irradiated with light at the dose of 21 J/cm^2^. The obtained results indicated a reduction in the expression of each of the mentioned genes. Changes in the metabolic profile of cells subjected to PACT show that fungi that are able to survive the therapy function in a significantly impaired manner [45]. These study results correspond directly to the observations made by the group of de Carvalho Leonel et al. [46]. They found out that PACT with the use of **1** not only reduced the number of *C. albicans* cells but also affected their growth kinetics [46]. The influence of PACT on the metabolism of *C. albicans* cells was also dependent to some extent on the fungal growth phase [47]. One study compared the effects of therapy on the *C. albicans* cells in lag (6 h) and stationary (48 h) phases. Fungal cells were exposed to **1** and irradiated with the light doses of either 130 J/cm^2^, 162 J/cm^2^, or 194 J/cm^2^. After the procedure, wrinkling and contraction of the cell membrane in both younger and older cells were noticed. In young cells, damage to intracellular structures was more visible. On the other hand, disturbances in extracellular polymeric substance (EPS) were observed only in stationary phase cells. Significant changes in the metabolome of the cells were also observed after the use of PACT. Cells in the stationary phase showed greater damage of polysaccharides and lipids while in the case of cells in lag phase proteins, and to some extent nucleic acids, were destroyed [47]. The use of PACT resulted in the prolongation of the lag phase and an alteration in the profile of the exponential phase after PACT, which may be related to the aforementioned changes in gene expression [46]. The scientists in the study also analyzed the relationship between the fungicidal potential of the dye and its concentration and the dose of light used in the experiment. The researchers used PS concentrations ranging from 0.01 to 0.05 mg/mL. Interestingly, the increase in concentration induced an increase in PACT activity to a limited extent. The best results were obtained with the PS concentration of 0.02 mg/mL and with the highest light dose (30 J/cm^2^), when 74% inhibition of biofilm formation was achieved [46]. In another experiment, the growth rate and viability of *C. albicans* cells were analyzed after the use of PACT. In line with the previously discussed studies, the use of sublethal PACT significantly impaired the fungal cell function [48]. First of all, a decreased integrity of the cell walls and greater susceptibility to oxidative stress were observed. After treatment, the cells were more sensitive to caffeine, hydrogen peroxide and sodium dodecyl sulfate. Greater sensitivity to fluconazole was also noted, which may be related to structural changes in the cell wall. Interestingly, the described changes were not observed in the next generations, and therefore, it can be concluded that PACT with the use of methylene blue does not inflict significant damage to the genetic material. It also raises an important question about the degree of penetration of PS molecules into the cell interior [48].

The efficiency of **1** in PACT is strongly correlated with this compound’s tendency to aggregate [49]. The aggregated form of the PS has a significantly lower bactericidal and fungicidal potential. From this perspective, counteracting this process is a significant technological challenge. Potential **1** formulations must prevent the aggregation phenomenon. Studies on the relationship between the solvent used and the effectiveness of therapy are currently being developed. One of the studies assessed the **1** behavior in the following media: water, physiological solution—NaCl 0.9%, phosphate saline buffer—PBS, sodium dodecyl sulfate 0.25%—SDS, and urea (1 M) [49]. With a rise of the **1** concentration, the electrostatic and hydrophobic interaction between molecules take place more often, resulting in a higher aggregate formation frequency. As a result of PS particles’ combination, dimeric forms arise, which show a lower ability to generate ROS (self-quenching phenomenon). Only the use of sodium dodecyl sulfate increased efficiency of the PDT in a statistically significant manner [49]. Moreover, there was no correlation between the length of dark incubation and the PACT yield using **1**. Extending the incubation time from 1 min to even 20 min did not increase the fungicidal properties of the PS. Similar conclusions can be drawn from other studies where different concentrations of **1** were tested [50]. In one of the experiments, in which a wide range of PS doses were used (from 5 to 60 μM), the optimal concentration was only 20 μM. In case of the light fluence evaluation, the highest dose of 60 J/cm^2^ led to the best fungicidal effects. The light was delivered in a single session or repeated over several sessions. No increase in PACT efficacy was observed after a repeated illumination. The authors suggested that the lack of efficiency increase with the simultaneous increase in concentration may be either due to a problem with the light penetration or because of the predominant but inefficient thermal mechanism [50]. However, in the light of other studies, it seems more likely that the main limitation is due to the aggregation of the planar-structured PS [51]. An assessment of the efficacy of PACT against fungi, especially *C. albicans*, is hampered by the lack of homogeneity of the data (Table 1). Depending on the parameters applied, the effectiveness of PACT with the use of **1** may vary from about 20% to over 99% reduction. An essential advantage of PACT is that it can be used against drug-resistant fungi. Interestingly, studies comparing the efficacy of **1** against fluconazole-resistant fungal strains compared to fluconazole-susceptible ones do not show a significant differences in activity [51]. A much more marked influence on the sensitivity of fungi exerts the composition of the cell wall, and above all—the composition of mannan that builds it, along with its hydrophobicity [52,110]. Based on these cell properties, two serotypes of *C. albicans* can be distinguished: A and B, which have different sensitivity to PACT and **1**. Rossoni et al. have used a dye at a concentration of 300 μM and the light dose of 26.3 J/cm^2^ [52]. After irradiation, log reductions of 0.49 for serotype A and of 2.34 for serotype B were observed. The frequency of different *C. albicans* serotypes varies for different populations and is difficult to predict. As the authors suspect, the difference in effectiveness of PACT is related to the permeability and integrity of microbial cells. The higher the cell permeability, the more effectively the PS can penetrate into the cell interior [52].

An important factor influencing the effectiveness of PACT against *C. albicans* and other fungi is the activity of multidrug efflux systems (MESs) [101]. Among wild fungi strains, overexpression of some classes of MESs is sometimes observed, including ATP-binding cassette (ABC), responsible for xenobiotic efflux out of the cell. This leads to the reduced fungicidal effectiveness of the PS. The activity of the transporter poses a significant challenge to PDT, and thus, one of the directions of PACT development is the search for efflux system inhibitors. One of the solutions may be the use of verapamil [101], although its use is not always effective [53]. In one study, the addition of either verapamil (at a dose of 50 μM) or sodium azide (at a dose of 1 mM) was evaluated when *C. albicans* was treated with **1**-mediated PACT. Each of the additives reduced the effectiveness of the therapy. While the effect of sodium azide seems to be clear due to its singlet oxygen quenching properties, the effect of verapamil is confusing. The authors suggest that it was related to calcium concentration, which may affect the effectiveness of PDT [53]. However, thus far, verapamil has a proven effect only on calcium channels in mammalian cells, and thus, such conclusions should be treated with caution. Moreover, subsequent studies, including in vitro studies not related directly to photochemistry, indicated that verapamil expresses some activity against *C. albicans* [111].

An interesting modification of the PDT using **1** is the addition of glucose solution to the protocol. There is evidence that *C. albicans* is able to detect the presence of glucose in the environment [101,112]. In the study, a solution of **1** in PBS was used in PACT towards *C. albicans* biofilm [54]. After exposure to 50 mM glucose (or PBS), incubation with the PS for 30 min, and subsequent illumination, a reduction of 85% in yeast viability was observed in the culture containing glucose compared to 70% without the glucose addition. It seems that adding glucose along with **1** to yeast suspension increases the uptake of **1**, thus increasing the effectiveness of PDT. However, de Oliveira-Silva et al. came to the opposite conclusions [55]. In their study, the effect of glucose on **1** uptake by *C. albicans* was negligible and did not affect the efficacy of the PDT. On the other hand, the researchers used a fivefold lower concentration (100 µM) of the PS and exposed the cells to glucose for 120 min (instead of 90 min) [55]. Chitosan was another carbohydrate that was investigated to influence the effectiveness of PACT using **1** [113]. Chitosan is a polysaccharide composed of β- (1–4)-linked D-glucosamine and *N*-acetyl-D-glucosamine, which expresses some antimicrobial properties [113]. Due to its interesting drug delivery properties, its combination with PS has been investigated, but thus far, only one significant study with chitosan-**1** has been reported. Despite the antifungal activity per se of the polymer, no additive activity or synergism was observed for combination of chitosan and **1** [56].

Another approach for improvement of the antifungal PACT is to add a surfactant to the formulation. Surfactants are compounds with certain antifungal properties and an amphiphilic nature. Thus far, the combination of **1** as a PS with one of the four different surfactants at sub-inhibitory concentrations was studied: cetyltrimethylammonium chloride (CTAC), *N*-hexadecyl-*N*-*N*’-dimethyl-3-ammonio-1-propane-sulfonate (HPS), sodium dodecyl sulfate (SDS), and Triton X-100 [57]. Before the PDT treatment, the yeast suspension was incubated for 60 min with the selected surfactant at the concentration of 0.3 µg/mL. The use of combined therapy was proven to be more effective than the use of either surfactant or PACT alone. The best results were obtained for **1** at 32 µg/mL with either CTAC or HPS, which allowed for complete killing the yeast [57].

### 3.2. Toluidine Blue O and New Toluidine Blue O

Toluidine blue O (tolonium chloride, **3**, Figure 3) has been known for over a hundred years as acidophilic metachromatic dye that selectively stains acidic tissue [114]. It is partially soluble in water and in methanol and has a high affinity for nucleic acids. Initially used mainly in industry, **3** was utilized in medicine as an antidote against heparin overdose and as a dye in histopathology [114]. It has also been used for some time in PDT as a PS, both in cancer therapy and in antibacterial therapy [115,116]. Interestingly, it was discovered that **3** in the presence of bacteria forms dimers (**4**, Figure 3), which are more photoactive. In comparison to **1** when tested on the cultures of *Staphylococcus aureus*, *Streptococcus pneumoniae*, *Enterococcus faecalis*, *Hemophilus influenzae*, *Escherichia coli*, and *Pseudomonas aeruginosa*, it turned out that **3** dimerizes more strongly than **1**, while showing greater activity [115,116].

Tests of **3** have been carried out in antifungal PACT as well, mostly towards *Candida* spp. and *Trichophyton* spp. inactivation. Experiments conducted thus far have provided a fairly broad overview of the activity of this PS. Despite relatively few studies on the efficacy of **3** against fungi other than *C. albicans*, different sensitivities of individual species can be clearly observed [58]. The fungi *Metarhizium anisopliae* and *Aspergillus nidulans* were tested in PACT, and both were inactivated in 99.7% in optimal conditions. A delay in sprouting of conidia was also observed among the surviving fungi. The experiment assessed whether washing the conidia before irradiation affects the effectiveness of PACT. The result was different for individual species: in the case of *A. nidulans*, a huge drop in the effectiveness of the therapy was observed, while in the case of *M. anisopliae*, only a slight difference was observed [58]. This indicates that fungal cell wall structure among diverse species may affect the way the PS binds or penetrates. One of the key advantages of **3** in PACT against fungi is its effectiveness against biofilms, which are more resistant to therapy. The use of **3** not only kills fungal cells but also impairs the functioning of the surviving structures [59]. The **3**-mediated PACT is capable of reducing the number of cells and filamentous form present in the *C. albicans* biofilm, as well as it limits the development of the biofilm even 24 h after application. A positive correlation was observed between the **3** concentration and the effectiveness of PACT with the 0.1 mg/mL **3** resulting in 61% decrease of cell viability. Interestingly, **3** was effective regardless of the stage of biofilm development [59]. The activity of **3** was confirmed in another experiment using lower doses [60]. The use of PACT resulted in an increase in ROS production in 11.43, 6.27, and 4.37 times for the concentrations of 0.01, 0.02, and 0.05 mg/mL, respectively, in comparison to the dark phase. For this study, the highest activity of PACT was at a concentration of 0.01 mg/mL and irradiation with the dose 40 J/cm^2^ of light. Under these conditions, *C. krusei* growth inhibition by 70% was observed [60]. A study conducted by Wiench et al. showed the usefulness of **3** in antifungal PACT [61]. In this experiment, plates prepared with methylmethacrylate polymer, which is frequently used in prosthetics, were immersed in a fungal suspension (either *C. albicans*, *C. glabrata*, or *C. krusei*) and then subjected to **3**-mediated PACT. After 60 s of incubation followed by 30 s irradiation, it turned out that this procedure was effective against each strain, and an increase in activity was observed with an increasing light dose (dose equal 24 J/cm^2^ at the light-source power 400 mW) [61]. After initial in vitro tests against *Trichopyton rubrum*, **3** also found an application in the treatment of onychomycosis [62]. The use of PS in the in vitro model at a concentration of 20 µg/mL after 30 min irradiation with 200 J/cm^2^ light led to complete eradication of fungi. The promising results encouraged researchers to apply the **3** formulation in a patient with confirmed onychomycosis. The use of three PACT sessions over the week led to a significant improvement in nail morphology, confirmed in a 6-month follow-up [62]. The effectiveness of PACT and cyclopiroxolamine against *T. rubrum* dermatophytes was also experimentally compared. It was shown that the use of **3** at the concentration of 10 mg/L and light at the intensity of 48 J/cm^2^ was undoubtedly more effective than the popularly used in medicine cyclopiroxolamine [63]. PACT was fungicidal or inhibited 98% of fungal growth depending on the strain tested. For comparison, the MIC values reported for the antifungal activity in this study were 2.0 mg/L for 90% of the strains. An apparent increase in the concentration of reactive nitrogen species (RNS, mainly ONOO^−^ and NO^•^) and ROS after the use of PACT confirmed their key role in the fungicidal mechanism. The presence of both RNS and ROS suggests that **3** interacts with the surrounding biomolecules. Increased availability of transition electrons, along with the concentration of RNT and ROS, causes oxidative and nitrosative damage in the fungal cell [63].

### 3.3. Combination of Phenothiazines with Antifungals

An interesting and promising modification of PACT is its combination with antifungal therapy. It gives both methods advantages and reduces the effective concentration of PS and antifungal concentration. Since the mechanism of action of antifungals and PACT on bacteria is completely different, both treatments can have an adjuvant effect. Antifungals and PSs have different molecular targets as a result of increasing critical and lethal damage to fungal cells [48,117,118,119]. Despite the undoubted advantages of PACT, its limitations should be kept in mind, especially the limited light permeability through tissues. While superficial infections are relatively easy to eradicate with PACT, deep tissue infections pose a serious challenge [20,44,77]. In such cases, a combination of the two approaches can yield exceptionally good results [120,121]. Effectiveness of the combination of antifungal PDT with the conventional antifungal therapy has also been confirmed by studies conducted on wax moth *Galleria mellonella* infected with *C. albicans* [65]. In the applied protocol, **1** and its combination with fluconazole were used. Moths that were infected with fungi as larvae survived longer after applying PACT, but this effect was only seen in animals infected with the wild strain. The subjects infected with the fluconazole-resistant strain did not show statistically significant difference in life expectancy. The combination of fluconazole and PACT significantly extended the lifetime of moths infected with a fluconazole-resistant strain. The authors suggest that this may be related to the antifungal-induced oxidative stress, thus enhancing the formation of ROS [65]. Other quite surprising study results were reported in 2016 by Ferreira et al. [66]. In this experiment, *C. albicans* suspension was treated with fluconazole followed by PACT using **1**. The use of the antifungal resulted in delaying the complete inactivation of the yeast [66]. These results are in obvious opposition to those quoted earlier, but the authors do not attempt to explain the phenomenon unequivocally. One of the mechanisms that sensitize fungi to antifungals is the destruction of extracellular polymeric substance (EPS) of biofilm by PACT. Even partial removal of external polymers can increase effectiveness of the classic antifungal therapy. Increased effectiveness of fluconazole has been observed with the use of **3** against *C. albicans* plankton [64]. Importantly, the increased sensitivity of fungi to antifungals was transient and lasted for about two hours. This observation indicates that the combination of PACT and an antifungal therapy requires an appropriate study design and treatment regimen. Sensitivity of the fungi to antifungals was also significantly lower in the case of biofilms. Only a 3-log reduction was observed when PACT with fluconazole were used against *C. albicans* biofilm. Complete killing of the biofilm was only observed when the photodynamic therapy was combined with capsofungin [64].

Outstanding results were also obtained with the combination of PACT and a wide range of antifungals against five strains of *Fusarium* spp. and five strains of *Exophiala* spp. in both their plankton and biofilm forms [67]. In this study, itraconazole, voriconazole, posaconazole and amphotericin B were used in combination with **1** as a PS. As a result of the action of PACT alone, the number of fungi was reduced by up to 6.4 and 5.6 logs, for plankton and biofilm, respectively. The earlier use of PACT allowed to obtain MIC values up to 64 times lower for each of the above-mentioned antifungals [67]. The authors suggest that the phenomenon may be due to the damage sustained by the outer cell barriers during the PACT.

### 3.4. Combinations of Phenothiazines with Nanoparticles

Intensive development of the PDT in recent years has included the search for new carriers for PSs. A promising area of photodynamic research are attempts to combine dyes with nanoparticles (NPs) which increase the selectivity of PS and/or efficacy of therapy. Regardless of the composition, NPs are a specific type of particles in sizes between 1 and 100 nm (with the surrounding interfacial layer) [122]. NPs have large, specific surfaces that can be modified in various ways [123]. Growth of a pathogen on the surface of the NP also increases its contact with the PS deposited there. A higher concentration of an active substance at the site of infection significantly upsurges the effectiveness of PACT and, at the same time, reduces the systemic side effects [124]. The combination of NPs and a PS allows the modification of their properties in a relatively easy way. It is possible to obtain conjugates releasing the PS in a specific, controlled manner or directly penetrating the bacterial cell [125,126,127]. Despite the unique and highly desirable properties of NPs, their combinations with phenothiazines in PACT against fungi have been very poorly studied and are limited to gold NPs (AuNPs) [128]. In the case of AuNPs, the so-called photothermal activity is characteristic. The AuNPs, excited under the influence of light, are able to effectively release the energy to the environment in a non-radiative manner—by emitting heat. This property has been used mainly in the research of photothermal cancer therapy [129].

Sherwani et al. prepared spheroidal NPs with an average size of 10–20 nm AuNPs using the *Aloe vera* extract [83]. Colloidal AuNP suspension with a slightly alkaline pH produced negatively charged particles which were then electrostatically attached to positively charged PSs (**1** or **3**). In the in vitro activity study against *C. albicans* and *Candida glabrata*, solutions or suspensions at the concentration of 200 μg/mL (AuNP, **1**, **3**, AuNP-**1**, AuNP-**3** and AuNP-**1** + AuNP-**3** formulations) were used. After half an hour of incubation, the suspensions were irradiated for 20 min. Analogous studies were also carried out in vivo on mice, whose skin was infected with *C. albicans*. Both in vitro and in vivo studies confirmed the effectiveness of the combination of **3** and **1** with AuNPs against fungal infection. In the histopathological examination, significantly fewer *C. albicans* cells were observed after the application of PACT. The best results were achieved when AuNPs were used simultaneously with **3** and **1**, although the authors did not clearly explain the potential synergistic mechanism [83]. Similar results were obtained in another experiment in which AuNPs prepared with the traditional method of HAuCl_4_ reduction were used [96]. The NPs with the average size of 21 nm were combined with **1**. Their activity at a concentration of 20 μg/mL was tested against *C. albicans*. After irradiation with 660 nm light at the dose of 38.2 J/cm^2^, reductions of 63% (**1**) and 82% (AuNPs-**1**) were observed. Results of a fluorescence spectroscopy analysis indicated that the type I photodynamic process was the dominant mechanism of the conjugate activity [96].

## 4. Xanthenes

### 4.1. Erythrosine

**Figure 4:**
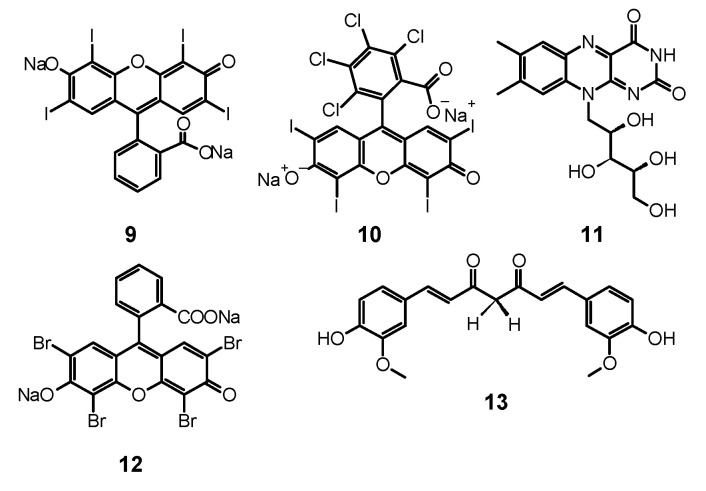
Chemical structures of (**9**–**13**).

Erythrosine (**9**, Figure 4) is a water soluble xanthene derivative that absorbs light in the blue and green region of visible spectra with a maximum absorption at λ_max_ = 530 nm [130]. Moreover, it presents high singlet oxygen quantum yield up to Φ_Δ_ = 0.63 [130,131]. Different fungal growth inhibition can be shown by **9** dependent on the PS concentration, light dose, and the form of fungal culture. A significant difference was observed between planktonic cultures and biofilm of *C. albicans* and *C. dubliniensis* using **9** as PS. The concentration of 3.12 µM was sufficient to eradicate the plankton form of both species. However, even 400 µM used on biofilm did not suffice, as the observed reduction was 0.74 log for *C. albicans* and 0.21 log for *C. dubliniensis* [130]. De Figueiredo Freitas et al. compared the impact of different light sources, LED (532 nm; 42.63 J/cm^2^) and laser (660 nm; 26.3 J/cm^2^) on excitation and photodynamic activity of **9** (400 μM) against *C. glabrata* biofilm. After the first procedure of PACT, fungal growth reduction achieved by using laser and LED was 3.36 log and 4.64 log, respectively. In the same study, the authors investigated the influence of multiple subsequent applications of photodynamic treatment. The fourth photodynamic treatment after a few days resulted in a fungal growth reduction of 5.94 log. Considering these results, a conclusion can be drawn that one of the ways to deal with much higher resistance of biofilms is the repeated PACT protocol. According to the authors, each photodynamic session weakened the biofilm surface structure more, leading to its enhanced susceptibility to PACT [132]. Additionally, in the experiments performed by Costa et al. with **9** (400 μM) irradiated with LED light source (532 nm) at the dose of 14 J/cm^2^ a reduction of 35% in the adherence of *C. albicans* to human buccal epithelial cells were shown [133]. This phenomenon directly reduces the ability of yeast to form a biofilm. Simultaneously, no damage to epithelial cells was detected [133]. Interesting results using **9** with blue dental LED (450 nm; 28.8 J/cm^2^) were presented by Silva et al., who achieved total reduction of planktonic and biofilm phenotypes of *C. albicans*, including a fluconazole-resistant strain [134]. However, in the in vivo experiments, no significant antifungal effect was observed. Authors suggested that the lack of activity is caused by high concentrations of PS, which led to aggregate formation, i.e., dimers, that reveal low singlet oxygen formation ability. Another factor responsible for the activity decrease in the animal model was poor diffusion of ER into the tissues [134].

Tomé et al. studied the photodynamic potential of **9** in the presence of sucrose. They reported that cultivation of the fungal biofilm with sucrose resulted in decreasing PACT efficacy against *C. albicans*. An increase in biofilm growth was observed when *C. albicans* was cultured with *S. mutans* in the medium with sucrose. Such complex bacteriofungal biofilm showed significant resistance to PACT treatment. Authors suggested that it was the result of an improved exopolysaccharide matrix production due to an easy access to the substrate—sucrose. Exopolysaccharide matrix made the biofilm more viscous and, in consequence, affected the accessibility of PSs to the target [135]. The effect of the co-culturing of two species was also investigated by Palma et al., who tested PACT potential against *C. albicans* and *S. sanguinis* biofilm [136]. The use of **9** (400 µM) and LED light (532 nm, 42.63 J/cm^2^) resulted in the reduction of *C. albicans* biofilm alone equal 1.07 log, whereas for mixed biofilm only 0.39 log [136]. The same group also reported a limited potential of growth reduction of *C. albicans* and *C. tropicalis* biofilms by PACT treatment with 200 µM **9** and green light (532 nm, 237 mW/cm^2^, 42.6 J/cm^2^) [137].

### 4.2. Rose Bengal

There has been an increased interest in Rose Bengal (**10**, Figure 4) as a PS in PACT against fungi in recent years. It is a compound from the xanthan group that is anionic and hydrophilic, characterized by the ability to absorb light in the NIR range. Thus far, it has been used in diagnosis of liver diseases and as a candidate for a drug for melanoma using the sono-photodynamic therapy approach (SPDT) [138]. Ambiguous results from studies on PACT using **10** against fungi were obtained. In a study conducted by da Silva et al., **10** (12.5 μM) and light at 16.2 J/cm^2^ were used against mixed *C. albicans* and *Bacillus atrophaeus* biofilm [139]. After irradiation, a reduction in CFU/mL of about 30% was observed, but the same rate of CFU reduction was noticed for control sample [139]. However, the reason for the low effectiveness of the therapy could be too short pre-irradiation time of only 5 min or insufficient light dose. In another study against planktonic and biofilm *C. albicans*, a comparatively lower dose of PS (40 µM) and a significantly higher dose of light (95 J/cm^2^) were used [104]. The results obtained were also not excellent; only a reduction by 1.97 log was achieved in planktonic culture and less than 1 against biofilm [104]. The relationship between concentration and photodynamic activity of **10** is widely discussed in the work of Freire et al. [140]. The PS was used there at a dose of 0.78, 1.56, 3.12, 6.25, 25, 50, 200, and 400 μM, where the highest activity towards a planktonic form of *C. albicans* was obtained at doses of 12.5, 25, and 50 μM (reduction by over 5 log), but no activity against biofilms was observed [140]. In addition, **10** was also used to treat onychomycosis caused by *T. rubrum* [141]. Effectiveness of the therapy was tested after 30 min. of incubation followed by irradiation with 532 nm light at fluences of 68, 133, and 228 J/cm^2^ and the PS at the dose of 140 µM. Optical microscopy confirmed an uptake of **10** by fungal cells. The fungicidal effect higher than 80% was achieved only by exposure to the light dose of 228 J/cm^2^ [141]. PACT against corneal fungal isolates—*Fusarium solani*, *Aspergillus fumigatus*, and *C. albicans* also led to promising results [142]. Moreover, **10** at a concentration of 0.1% and light irradiation at a fluence of 5.4 J/cm^2^ were used, and a strong growth inhibition was observed for each of the tested strains. However, it should be emphasized that the applied research method was significantly different from the others analyzed in this review, and the obtained results are difficult to unambiguously compare with the others [142].

Enhancement of the PACT method can be achieved by using nanoparticles. Maliszewska et al. investigated biogenic AuNPs in a mixture with **10** against planktonic and biofilm forms of *C. albicans*. AuNPs enhanced the efficacy of **10**. Exposure of biofilm (500–780 nm xenon lamp; 95.4 J/cm^2^) for 30 min resulted in a reduction in CFU of 0.6 log and 1.53 log for **10** and **10** with AuNPs respectively. Authors claim that AuNPs may also change the mechanism of photosensitization from type II to type I and facilitate the interaction between the cell membrane components of a fungal cell and PS. Moreover, it was found that gold nanoparticles improved the accumulation of **10**, as after 120 min of incubation with the cells, followed by washing, the amount of PS within the *C. albicans* cells reached 58% of the initial quantity compared to 45% without using AuNPs [143].

A study of **10** in combination with antifungals was also carried out. The in vitro tests proved the sensitivity of one of the most common dermatophytes, *T. rubrum*, to the combination of **10** with clotrimazole [144]. Exposure to clotrimazole followed by **10**-mediated PACT allowed to significantly reduce the exposure time in PACT. Initially, the parameters for the PACT alone included the use of 24 J/cm^2^ light and **10** at a concentration of 140 μM, and this allowed for a complete reduction of the viability of the tested model fungi. The addition of a sublethal dose of clotrimazole to the regimen resulted in a halved pathogen irradiation time. The authors emphasized that the sequence was crucial in the applied protocol: firstly, the antifungal agent, and secondly, the PACT [144]. The activity of xanthenes is summarized in Table 2.

## 5. Curcumin

Nowadays, many studies have been performed to obtain PSs of the best quality [145]. One of such compounds may be curcumin (**13**, Figure 4), a natural polyphenolic compound from turmeric. It becomes a more and more popular PS used in PACT as an alternative treatment of fungal infections in the face of increasing antimicrobial resistance and recommended for susceptible patients as HIV-infected and those under chemotherapy. Owing to its bioactive properties, it is considered an antibacterial and antifungal agent, which has been tested in many experiments [146]. Even in micromolar concentrations, **13** reveals phototoxic potential after irradiation with light of the wavelength 300–500 nm [145,147,148].

Scientists compared also the effectiveness of **13** mediated PACT against planktonic and biofilm forms of pathogens. Dovigo et al. evaluated PDT mediated by **13** against *C. albicans*, *C. tropicalis,* and *C. glabrata*, both in planktonic and biofilm forms [149]. Suspensions of all pathogens were treated with **13** and exposed to LED, which promoted a substantial antifungal effect against the planktonic form of the yeasts. At the concentration of **13** of 40 μM and at the light dose of 18 J/cm^2^, both the metabolic activity of biofilms of all species evaluated and their biomass were significantly reduced. The researchers came to the conclusion that low concentrations of **13** can be sufficient to inactivate *Candida* isolates when combined with light irradiation [149]. Sanitá et al. investigated the effectiveness of **13**-mediated PACT combined with LED light against biofilms of *C. dubliniensis* [150]. They tested three isolates from HIV-positive patients and one reference strain. Samples were treated with **13** of three concentrations (20.0, 30.0, and 40.0 μM), incubated in the dark for 20 min and exposed to 5.28 J/cm^2^ of LED light. Additional samples were treated either with **13** or LED light only, and to the control samples, neither **13** nor light was applied. Cellular uptake of **13** by yeast cells and drug penetration through the biofilms were evaluated with confocal laser scanning microscopy. Antimicrobial PDT mediated by **13** reduced considerably the metabolism of the biofilm of *C. dubliniensis*, although a longer preirradiation time was necessary for photosensitizing the biofilm than for planktonic cultures [150]. Moreover, **13** turns out to be beneficial for treatments combining PDT with conventional antifungal therapy. Taiwanese researchers developed a treatment against the very common human pathogen *C. albicans* [151]. They applied **13** and blue light, and additionally, they examined this combination with fluconazole treatment. They compared the effects of fluconazole therapy and found a higher decrease of the viability of planktonic *C. albicans* than that of its adherent form in biofilm. PACT turned out to enhance the effectiveness of eliminating *C. albicans* biofilms and particularly in combination with fluconazole, 5 μM of **13** and 9 J/cm^2^ of blue light being adequate for elimination of biofilms. While fluconazole eradicates the yeast form, **13** mediated PACT is directed at fighting biofilms. Therefore, the combination of these three components would inhibit the growth and virulence of *C. albicans* and prevent drug resistance [151]. Some research has been carried out to investigate the antifungal mechanisms associated with **13** mediated PACT at the level of genes. Ma et al. studied gene expressions related to *C. albicans* biofilm formation in vitro to find out alterations in biofilms treated with PACT [152]. They confirmed the potential of **13**-mediated PACT to inactivate *C. albicans*. They also noted that expression of genes involved in biofilm formation was downregulated after applying a treatment that inhibited hyphal growth, caused cell separation and led to defective biofilm formation. What is interesting, light irradiation itself, without **13**, resulted also in downregulation of the genes [152]. Jordão et al. investigated whether the oxidative stress induced by PACT affected the expression of *C. albicans* genes associated with adhesion and biofilm formation (ALS1 and HPW1) and oxidative stress response (CAP1, CAT1, and SOD1) [153]. In the PACT two photosensitizing agents (PSs)—Photodithazine^®^ (chlorin derivative—**38**, Figure 9) (100 and 200 mg/L) or **13** (40 and 80 μM) were used in combination with LED (37.5 J/cm^2^ or 50 J/cm^2^). The gene expression was quantified with Reverse Transcription-Quantitative Polymerase Chain Reaction (RT-qPCR) and specific primers for the target genes. It turned out that both Photodithazine^®^ and **13**-mediated PACT affected the expression of all five *C. albicans* genes evaluated. Both the PACT mediated by LED-associated PSs Photodithazine^®^ and **13** promoted a reduction in the expression of the five *C. albicans* genes evaluated. The expression of genes related to adhesion and biofilm formation (ALS1 and HWP1) and also genes responsible for the oxidative stress response were reduced. Analysis of the results led to an observation of the decrease of the virulence and the adaptability to oxidative stress of *C. albicans* after PACT procedure, irrespective of the PS applied [153]. Many scientists focused their research on the concentration levels of PS, its formulations, variations of dosimetry in PACT and correlation with antifungal activity, and effectiveness against various species of fungi. Andrade et al. performed studies to assess the effects of pre-irradiation time (PIT) on **13**—mediated PACT against planktonic and biofilm cultures of *C. albicans, C. glabrata*, and *C. dubliniensis* [154]. The cell viability of all *Candida* species which had been maintained in contact with 40.0 mM **13** was highly reduced after 20 min of PIT. Planktonic cultures showed higher susceptibility than biofilm counterparts. Their inactivation did not depend on PIT, whereas PIT-dependence of the biofilm cultures varied among the species tested [154]. Scientists undertook research on the use of PACT against *T. rubrum*, the most common dermatophytic fungus inducing cutaneous infections [155]. They examined **13** as the PS in both free and nanoparticle (**13**@NP) form. Nanocarriers were silane derived sol–gels made from tetramethoxysilane (TMOS), combined with chitosan and polyethylene glycol, used for stabilization of **13**, its delivery, and solubility improvement. After application of optimal conditions of 10 μg/mL of PS with 10 J/cm^2^ of blue light at a wavelength of 417 ± 5 nm, ROS and RNS were generated and resulted in eradication of fungus. The reactive radicals did not tend to develop resistance and exerted their effects locally. Thus, **13** incorporated into NPs led to greater NO^•^ production and increased apoptosis of fungal cells, revealing more favorable properties than free **13**. It is worth noting that the preferential accumulation of **13**@NPs was observed within the hair follicles which are the clinical site of *T. rubrum* infection [155]. Carmello et al. carried out research on the potential of sublethal PACT using **13** and blue light at the wave length of 455 nm [156]. They aimed at generation of ROS in the form of singlet oxygen and DNA damage of *C. albicans*. Surprisingly, both **13**-mediated PACT and light alone indicated substantial DNA damage of *C. albicans* in comparison to the negative control. Similarly, the considerably higher intracellular ROS formation was observed for the group treated only with light. Nevertheless, PACT in comparison to blue light alone brought about extensive DNA damage, and its repair was inhibited by the use of **13**, which appeared to be the mechanism of the antifungal activity of the treatment procedure [156]. Da Silva et al. investigated *C. albicans* biofilms in artificial bone cavities treated with PACT mediated with 450 nm blue LED in combination with **13**, using fluorescence spectroscopy [157]. They also searched for a relation between the effectiveness of the photodynamic treatments and the fluorescence spectroscopy images. Forty artificial bone lesions in bovine bones were inoculated with standard suspensions of *C. albicans* to form biofilm. The following four experimental groups were separated into a control group, a group irradiated with LED for 5 min, a group where the cavities were filled with a solution of **13** for 5 min, and a group subjected to PACT mediated treatment including light and **13**. Samples were collected from the bone cavities after treatments and were seeded to obtain the colony-forming units (CFUs). The images of spectra fluorescence of the specimens were prepared before and after each treatment. The eradication of *C. albicans* in the group treated with PACT mediated procedure including light and **13** was the most significant, and the fluorescence images correlated with the microbiological results [157]. Dovigo et al. studied the association of **13** with LED for the photoinactivation of *C. albicans* [158]. They selected the investigation protocol after testing nine **13** concentrations and after exposition to different fluences of LED. In comparison with the control group, a considerable reduction in *C. albicans* viability was noted after PACT for both planktonic and biofilm cultures. After illumination of **13** with blue LED light, it appeared to be an effective photosensitizing agent to fight *C. albicans*, in both planktonic and biofilm form. The presence of **13** in the surrounding media was necessary for the efficient photodynamic treatment. The incubation time of 20 min enhanced PACT effectiveness against biofilms in comparison to 5 min. The short periods of irradiation proved to be sufficient for photoactivating the dye and exerting the phototoxic effects. Despite PACT phototoxicity towards macrophages, the therapy affected more the yeast cells than the defense cells [158]. Dovigo et al. examined the **13** mediated photoinactivation of *C. albicans* in a murine model of oral candidiasis to confirm its effectiveness in vivo [159]. Forty mice orally inoculated with *C. albicans* received topical **13** (20, 40 and 80 μM) after five days and were subjected to illumination with LED light. The applications of **13** or light alone were tested. All exposures to **13** with LED light resulted in a substantial drop in *C. albicans* viability after PACT, whereby the most encouraging results were obtained with the use of curcumin at 80 μM and illumination with 37.5 J/cm^2^. Moreover, this approach did not harm the host tissue of the animals [159]. Quishida et al. assessed the effectiveness of **13** mediated PACT on multispecies biofilms of *C. albicans*, *C. glabrata*, and *S. mutans* [160]. A total of 480 acrylic samples were incubated with bacteria and yeast for 24 or 48 h. For the photodynamic reaction, three concentrations of **13** (80, 100, and 120 μM) were applied and LED light. Some samples were treated with **13** or LED light only, control samples were treated neither with **13** nor irradiated. After PACT, CFUs were measured. Cell metabolism and the total biofilm biomass were examined. It turned out that both 24 and 48 h biofilms were susceptible to PACT mediated with **13** in the concentrations studied and light dose of 37.5 J/cm^2^, although the 24 h biofilm was more sensitive than the 48 h biofilm. For the 48 h biofilm, the decrease of fungal cell viability was noted for the highest concentration of **13** only. PACT also decreased metabolic activity and total biomass of the biofilms evaluated [160]. Pellissari et al. investigated the cytotoxicity of **13** mediated PACT towards human keratinocytes co-cultured with *C. albicans* [161]. They performed PACT on the experimental group. Moreover, the group subjected to LED irradiation only, the group with **13** solution not subjected to illumination, and the group neither including curcumin nor irradiated were examined. Additionally, keratinocytes and fungus cultures placed separately were a control group, not subjected to the PACT procedure. After PACT, the growth inhibition of keratinocytes and *C. albicans* was noted in all tests, which made it regarded as slightly to moderately cytotoxic. Groups irradiated only or treated with **13** only lowered the cell metabolism but did not inhibit the growth of *C. albicans*. Moreover, no impact of the cell interaction in co-culture was observed on the metabolism of keratinocytes and *C. albicans* [161]. Sakima et al. developed a formulation based on polymeric NPs to improve water solubility of **13**, applied as a PS in the PACT procedure against oral candidiasis induced by *C. albicans* in mice [162]. They synthesized anionic and cationic nanoparticles from poly-lactic acid and dextran sulfate. They applied free **13** or **13** encapsulated in NPs to murine tongues. Then they irradiated them with blue light for five consecutive days. *C. albicans* was recovered for further tests. Nystatin was used as a positive control and caused the greatest elimination of the fungus, followed by **13** mediated PACT. Anionic nanocarriers with **13** did not reveal antifungal potential, whereas cationic formulation eliminated the pathogen even without exposure to light. Observed DNA damage was related to fungal infection, not the treatments. Moreover, PACT with free **13** caused expression of specific cytokeratines of tongue epithelium—CK13 and CK14 similar to that of healthy animals, a result not observed for nystatin [162]. The activity of curcumin is summarized in Table 3.

## 6. Porphyrins

### 6.1. First Generation Photosensitizers

#### 6.1.1. Porfimer Sodium

**Figure 5:**
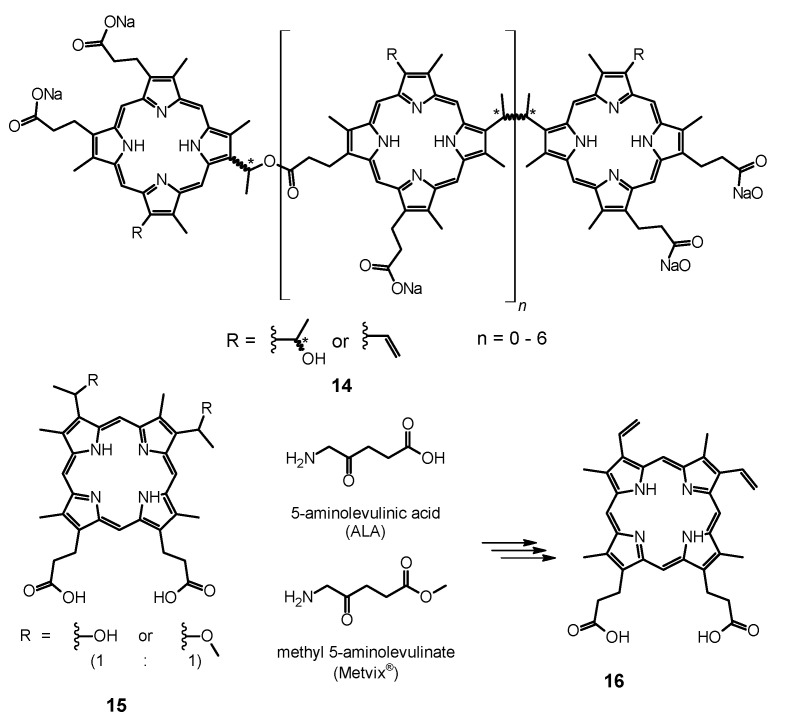
Chemical structure of **14**–**16**.

Bliss et al. performed a study aiming to assess the effectiveness of PACT with porfimer sodium (Photofrin^®^, **14**, Figure 5) against *Candida* species [163]. The authors compared the in vitro susceptibility of *C. albicans*, *C. krusei*, and *C. glabrata* to such therapy. Important findings included the fact that **14** induced photokilling in a dose dependent manner upon irradiation. Moreover, the internalization of the PS into the fungal cells was much lower in blastoconidia form as compared to filamentous form (hyphae). Additionally, it was noted that the take up of the PS depended on the medium in which the cells were grown. Importantly, the susceptibility to PACT was much lower in case of *C*. *glabrata* than for *C*. *albicans* or *C*. *krusei*. It was mostly associated with absorption of the PS which was proven using fluorescence microscopy [163]. The same PS was used in a report by Chabrier-Roselló et al., who investigated in vitro its effects exerted on *C*. *albicans* [164]. The authors found that after the application of the PS, it was not removed from the fungal cells; however, the photosensitivity decreased upon addition of the serum to the culture. Fungus was susceptible to photodynamic action both in its planktonic form, as well as in biofilm. Importantly, the *C*. *albicans* biofilms were found more susceptible to PACT than to amphotericin B chemotherapy. Moreover, no form of adaptive defense mechanism was noticed upon photodynamic treatment, unlike upon treatment with hydrogen peroxide, suggesting that the formed ROS—most notably singlet oxygen—do not induce cellular response to this mode of damage [164]. Mang et al. tested the photokilling of various *Candida* species—*C*. *albicans*, *C*. *glabrata*, *C*. *parapsilosis*, *C*. *krusei*, and *C*. *tropicalis*—induced by PACT with porfimer sodium [165]. The authors studied the optimal concentration of the PS, incubation time, and dosimetry for efficient PACT. These conditions were then used on Amphotericin B- or fluconazole-resistant clinical isolates from AIDS patients—*C. albicans*, *C. glabrata*, *C. guilliermondi*, *C. parapsilosis*, and *C. krusei*. It was found that the resistant strains were equally susceptible as their reference counterparts [165].

#### 6.1.2. Photogem^®^

Dovigo et al. researched the possible use of Photogem^®^ as a PS in PACT towards *C. albicans*, *C. dubliniensis*, *C. tropicalis*, and *C. krusei* [166]. Upon a series of experiments utilizing different concentrations and light doses, differences in susceptibility of various strains were observed. Importantly, the viabilities of all the fungi were reduced, but unlike other *Candida* species, eradication of *C*. *krusei* could not be achieved [166]. The studies were continued, as the same group tested Photogem^®^ photokilling efficacy against *C*. *albicans* and *C*. *glabrata* reference strains, as well as clinical isolates of these species which expressed fluconazole resistance [121]. It was found that different strains of a species showed significant differences in susceptibility to PACT, with fluconazole resistant strains being less affected. Moreover, *C*. *glabrata* was less sensitive than *C*. *albicans* to the same treatment protocol used, as well as the microorganisms in biofilm form were more resistant to photoinactivation than their corresponding planktonic forms [121]. This topic was continued later, as Mima et al. prepared artificial dentures inoculated with either *C*. *albicans*, *C*. *glabrata*, *C*. *tropicalis*, *C*. *krusei*, or *C*. *dubliniensis* and evaluated the inactivation of the microbes using PDT with Photogem^®^ sensitization [167]. Among the tested strains, *C*. *tropicalis* was shown to be the most susceptible with 3.99 log reduction, and the most resistant, *C*. *dubliniensis* with 1.73 log reduction obtained [167]. Photogem^®^ was also studied as a potential PS in vivo for oral candidiasis in a murine model [168]. After infecting the mice with *C*. *albicans*, the animals were subjected to PDT protocol using either blue (455 nm) or red (630 nm) light. As the *Candida* infection was superficial, there was no significant difference in reduction of cell viability which was in the range of 1.0–1.6, depending on the PS concentration used. The best results were obtained for the concentration of 500 mg/L of Photogem^®^, as compared to the groups treated with 400 mg/L and 1 000 mg/L. Importantly, no changes in the mucosa were found in the histological assessment [168]. The same group reported then five cases of patients treated for denture stomatitis using Photogem^®^ PACT [169]. In all patients, *C*. *albicans* infection was identified, in one case along with *C*. *glabrata* and in one case with *C*. *glabrata* and *C*. *tropicalis*. After the PDT procedure, the condition of 4 patients improved, but in the follow-up, in three of the patients, recurrence of the condition was noted while only in one case eradication of *C. albicans* was observed [169]. The authors highlighted the limitations of the study, such as low number of cases and lack of control groups, and later reported a clinical trial aiming to assess Photogem^®^ PACT in comparison to nystatin treatment of denture stomatitis [170]. Each group consisted of 20 patients and was administered nystatin for 15 days or irradiated after sensitization with Photogem^®^ three times per week over the course of 15 days. The prevalent microorganisms causing the condition were *Candida* species with *C. albicans* considering the most prevalent. The results showed higher clinical success rates of nystatin (53%) than PACT (45%), but high recurrence rate was observed in both groups (75% and 78%, respectively). Authors suggest that the low efficiency of PACT could be due to the use of only one PS concentration and one light dose, optimization of which could result in higher efficacy [170]. On the contrary, a report by Silva et al. documented a case of treatment of toenails onychomycosis which were treated successfully in six PACT sessions using Photogem^®^ as a PS [171]. Importantly, the application of the PS solution was preceded by removal of the top layer of the nail using a drill and a specially designed device was used for illumination [171]. Romano et al. also studied Photogem^®^ [172]. It was used as a PS for photoinactivation of *C. albicans*; in this case, however, the authors focused on the modification of the PACT protocol by irradiating the cell cultures with low intensity light (up to 4 J/cm^2^) during their incubation with the PS. Such approach enabled only a slight increase in internalization of Photogem^®^ but resulted in dramatic increase of PACT that followed (8 or 17 J/cm^2^). Light-driven incubation and PACT caused a reduction of over 7 log while dark incubation and irradiation induced < 1 log reduction. This effect was probably caused by the increased permeability of the outer layers of the cellular membranes and thus different localization of the PS in the cell [172].

#### 6.1.3. Hematoporphyrin Monomethyl Ether

Another porphyrin-based PS of which antifungal PACT activity was assessed was hematoporphyrin monomethyl ether (**15**, Figure 5). Use of **15** enabled to reduce the in vitro viability of planktonic *C*. *albicans* by 7 logs when optimal irradiation conditions were applied [173]. Moreover, performing an analogous experiment against azole-resistant *C*. *albicans* strain was found just as effective. The analyses on the treated cells revealed that upon irradiation **15** induced DNA damage in the treated cells, photodegradation of proteins, as well as changes in the cell wall, membrane, cytoplasm and nucleus when the highest concentration of **15** was used [173]. These studies were later enhanced by assessment of PACT towards biofilm forms of reference *C*. *albicans* and its azole-resistant clinical isolate [174]. In this case, the same conditions that led to eradication of the planktonic cultures resulted only in partial photokilling of the fungal strains—2.47 and 3.26 log reduction for standard and azole-resistant *C*. *albicans*, respectively [174].

### 6.2. Second Generation Photosensitizers

#### 6.2.1. 5-Aminolevulinic Acid and Its Derivatives

5-Aminolevulinic acid (ALA) is a pro-drug used in PDT, as it is a natural amino acid which is a substrate in protoporphyrin IX (**16**, Figure 5) biosynthesis pathway. By its application, excessive amounts of **16** are produced which can act as a PS. It has already been proven to be a successful compound used in PDT against cancer cells, and its importance in PACT is on the rise. The review from 2010 by Qiao et al. summarized the initial case studies in which ALA was used to treat fungal infections caused by *C. albicans*, *T. rubrum*, and *T. mentagrophytes* [175]. Although the conclusions from the studies were encouraging, as use of ALA enabled antifungal PACT, it was associated with high recurrence rate, minor adverse effects, and overall low clinical success rate. Further studies were needed to optimize the conditions and PACT protocols to fully reveal the potential of ALA [176,177]. Calzavara-Pinton et al. investigated the efficacy of ALA photodynamic treatment of interdigital mycoses [178]. The examination revealed that the microbes responsible for the conditions were *C*. *albicans* (3 cases), *T. mentagrophytes* (4 cases), and *T. rubrum* (2 cases). Infections were treated using topically administered ALA which was irradiated with red light (75 J/cm^2^). Such treatment was performed up to four times at weekly intervals. It was noted that in four patients, recurrence of the infection was observed after 4 weeks after the last therapy session, which could be linked to the location of the infection or too mild irradiation protocol used [178]. The use of ALA was also reported for treatment of cutaneous granuloma caused by *C*. *albicans* [179]. The patient was treated first with itraconazole for 4 weeks, and then, ALA was topically applied and irradiated after four hours (630 nm, 360 J/cm^2^), which led to complete recovery. The authors highlight the lack of adverse effects of the PACT as compared to systemic antifungal treatment [179]. Lee et al. reported six cases of treating *Malassezia*-induced folliculitis [180]. Methyl 5-aminolevulinate was applied to patients’ lesions, and after three hours the PACT protocol (37 J/cm^2^) was carried out, which was repeated three times in two-week intervals. Improvement of the condition was observed in five cases, while in one of them no change was seen. The lack of effect was explained by the fact that the patient was an athlete and increased sweating might alter the microenvironmental conditions in the patient’s follicles [180]. Aspiroz et al. described the treatment of *Acremonium sclerotigenum* onychomycosis using methyl 5-aminolevulinate in three sessions of the PACT, which resulted in full recovery even after 12 months after the therapy [181]. The same group reported a case of a patient suffering from toe nail infection caused by *Scytalidium dimidiatum*, which was resistant to treatment with topical antifungal agents [182]. PACT using methyl aminolevulinate was implemented. After three weeks of therapy involving three therapeutic sessions, no presence of the fungus was detected in microbiological cultures. After 6 months, the pathogen was again detected, but it did not penetrate the nail and did not cause any clinical symptoms. Importantly, in both described cases, the used treatment did not cause any adverse effects, even though it was performed on elderly patients [182]. Hu et al., on the other hand, reported a case where chromoblastomycosis caused by *Fonsecaea monophora* was successfully treated with the ALA PACT combined with terbinafine chemotherapy [183]. Before PACT was implemented, the infection had been treated over prolonged period of time with itraconazole and fluconazole. Further, monthly treatment with oral formulation of terbinafine also yielded no clinical improvement. Only after repeated ALA treatment in two irradiation series (each taking place four hours a week for nine weeks) with simultaneous terbinafine administration, the full recovery was achieved. In the following in vitro studies, the *F*. *monophora* was found susceptible to each antifungal drug that was used by the patient—itraconazole, fluconazole, and terbinafine. This suggests that the in vitro effect cannot be simply translated to clinical practice and strongly depends on other factors affecting the drug efficacy in vivo [183]. The group continued their research on the topic and reported further five cases of *Fonascea* infections (one *F*. *nubica*, two *F*. *pedrosoi*, and two *F*. *monophora*) treated with simultaneous ALA PACT and antifungal chemotherapy with clinical improvements but full recovery only in three cases [184]. Each of the isolates was found susceptible to terbinafine, itraconazole, and voriconazole, as well as to ALA PACT alone. Combination of the PACT and chemotherapy against *F*. *monophora* gave no improved effectiveness in vitro, yet the in vivo combined therapy was significant. The authors noticed that simultaneous use of ALA and itraconazole potentiated the antifungal activity of all the components and hypothesized that itraconazole might induce changes in the fungal cell wall and thus the internalization of ALA was increased, resulting in a synergistic effect. Yi et al. studied the effectiveness of ALA towards the conidia of *F. monophora* inactivation in vitro [185]. It was observed that such approach was successful, but more importantly, ALA PACT acted in a protective way on RAW264.7 macrophages, and thus the fungicidal effect was potentiated [185]. The study reported by Shi et al. dealt with the problem of photoinactivation of *C*. *albicans* biofilms [186]. First, on the cultured biofilms of *C*. *albicans,* the optimal conditions for internalization of ALA were tested. It was found that biosynthesis of **16** in the fungal cells was not dependent on the ALA concentration, as application of the lowest (1 mM) and the highest (30 mM) concentrations of ALA resulted in the lowest amounts of intracellular formation of **16**. The most optimal in this case was 15 mM concentration with 5 h incubation. With such conditions, along with the highest light dose used (300 J/cm^2^), it was possible to inhibit the fungi by nearly 75% [186]. An in vivo study was performed to compare the effectiveness of **16** and **1** in PACT of vaginal candidiasis in mice [187]. Both the PSs reduced the number of CFUs of *C*. *albicans* (nearly 1 log reduction), and neither of them caused total eradication of the fungus, which was deemed beneficial, as *Candida* is one of the species constituting vaginal microbiota. Authors also observed a decrease in inflammatory response, as well as lack of reinfection over the course of 7 days. What should be noted is that **1** was used in tenfold higher concentration than **16** (100 and 10 µM, respectively) [187].

#### 6.2.2. Others

**Figure 6:**
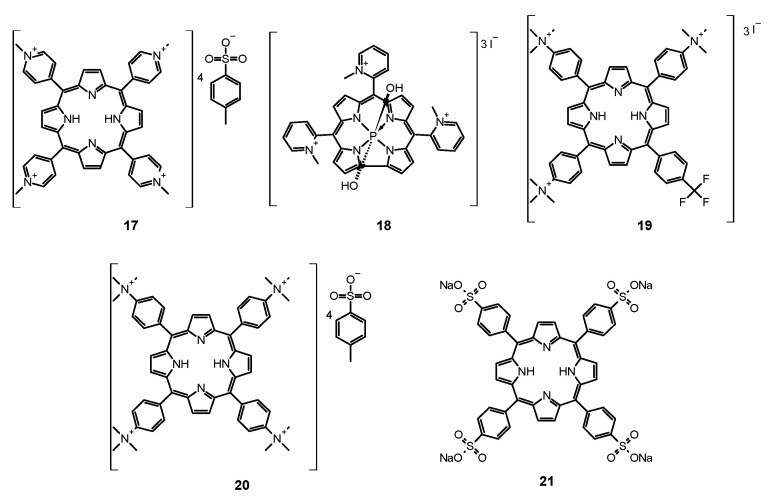
Chemical structures of **17**–**21**.

Chabrier-Roselló et al., investigated the susceptibility of various strains of *C*. *albicans* and *C*. *glabrata* in PACT using 5,10,15,20-tetrakis (*N*-methylpyrid-4-yl)porphyrin tetra (4-toluenesulfonate) (**17**, Figure 6) [188].

**Figure 7:**
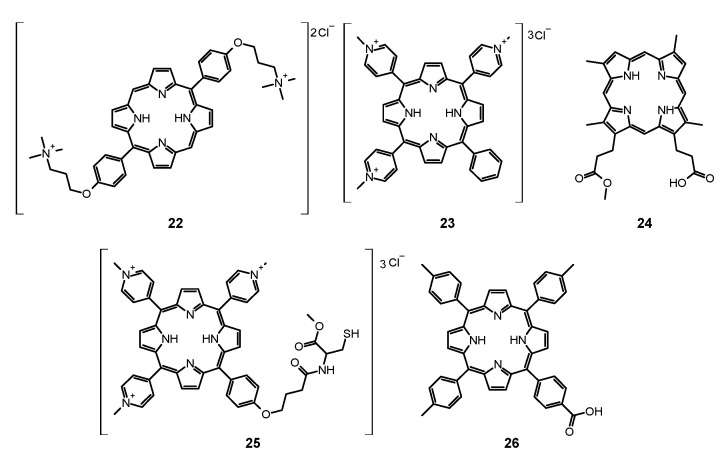
Chemical structures of **22**–**26**.

They noticed that *C*. *albicans* and *C*. *glabrata* were prone to photocytotoxic effect of this PS (over 3 logs reduction), while both were resistant to PACT with porfimer sodium. It was also found that respiratory-deficient strains of *Candida* present increased sensitivity to photodynamic treatment along with increased resistance to azoles. Authors theorized that the increased susceptibility of the mutant strains was due to changes in cell wall permeability and insufficient development of cell defense mechanisms against ROS [188]. This topic was further studied by the same group, as they evaluated the photodynamic action of **17** against various strains of *C*. *albicans*, *C*. *glabrata* and *Saccharomyces cerevisiae* [189]. Selected mutants of the species were used to pinpoint the genes or particular cellular components whose deactivation would lead to the best photocytotoxic effects. The authors concluded from the experiments that the inhibition of electron transport chain complexes III and IV during PACT offers the best efficacy of the induced photokilling [189]. Quiroga et al. also assessed the same PS towards *C*. *albicans* photoinactivation, which resulted in 5 log decrease of the microorganisms [190]. Bornhütter et al. studied the behavior of two PSs, **17** and 5,10,15-tris- (*N*-methylpyrid-2-yl) corrolato- (*trans*dihydroxo) phosphorus (V) (**18**, Figure 6), in *T. rubrum* and *Scopulariopsis brevicaulis* cell cultures in vitro [191]. The fluorescence of the compounds was monitored throughout 24 days of experiment, along with the singlet oxygen luminescence. The interesting findings include the fact that no difference was observed between various fungal species, but the PSs showed changes in photophysical properties over time [191]. These experiments were followed by a study assessing these two porphyrinoids in photodynamic inactivation of fungi responsible for onychomycosis, *T. rubrum*, *Trichophyton interdigitale*, and *S*. *brevicaulis*, in suspension and immobilized on a surface, as well as evaluation of anionic Eosin Y for PACT of the aforementioned fungi growing on surface [192]. The findings of this report included eradication of all the fungal species grown on surface by all three tested PSs. In the suspension cultures, PACT towards *T. rubrum* was the least successful with porphyrin derivative, while the corrole was efficient in its inactivation. In the cases of all the other fungal species, cell viability drastically decreased upon addition of the PS and light exposure. Interestingly, *S. brevicaulis* exhibited sensitivity to light alone, as the survival rate of the fungal cells dropped below 40% [192]. Cormick et al. compared the in vitro effectiveness of PACT of cationic and anionic porphyrins against *C*. *albicans* [193]. It was found that positively charged macrocycles, 5-(4-trifluorophenyl)-10,15,20-tris (4-trimethylammoniumphenyl)porphyrin triiodide (**19**, Figure 6) and 5,10,15,20-tetra (4-*N*,*N*,*N*-trimethylammoniumphenyl)porphyrin tetra (4-toluenesulfonate) (**20**, Figure 6), showed a better binding to the fungal cell wall than the anionic 5,10,15,20-tetra (4-sulfonatophenyl)porphyrin tetrasodium salt (**21**, Figure 6). The use of the same concentration of the PSs in PACT against *C*. *albicans* revealed the superior inactivation when cationic macrocycles were applied, in spite of higher singlet oxygen yields found for the anionic porphyrin [193]. Later on, the same groups evaluated the three cationic macrocycles: **19** and **20** which were tested before, as well as **17** [194]. All of the tested compounds revealed 5 log reduction of *C*. *albicans*. Additional experiments performed gave also some insight into the mechanism of antifungal action, as the porphyrins were strongly bound to the fungal cells and the inactivation occurred mostly by means of singlet oxygen production. Moreover, presence of molecular oxygen was necessary and no change in cell survival was observed in anoxic conditions [194]. In a study that followed, it was found that the cationic porphyrins tested interacted strongly with DNA but they had almost no effect on PACT efficacy [195]. In addition, **17** was also the subject of investigation by Davies et al. [196]. They assessed the use of this macrocycle in PACT of biofilms of reference cultures of *C*. *albicans* and *C*. *glabrata*, as well as clinical isolates: *C*. *albicans*, *C*. *glabrata*, *C*. *tropicalis,* and *C*. *parapsilosis*. Under the conditions applied (350–800 nm light irradiation for 58.5 J/cm^2^), no change in metabolic activity of *C*. *glabrata* was observed, whilst the other strains were affected with metabolic activity decreasing to nearly 65% of initial values. All the strains were susceptible to combined treatment using PACT with **17** and antifungal chemotherapy with miconazole even though some of them were resistant to one of the therapies when used alone. The combination efficacy could be additive or synergistic, depending on the *Candida* strain treated [196]. These findings are in good correlation with the results obtained by Snell et al., who showed that certain antifungals—especially miconazole—increased the effectiveness of PACT performed with **17** and **1** as well [197]. A comparison between two similar porphyrins was reported involving the 5,10,15,20-tetrakis [4-(3-*N*,*N*-dimethylaminopropoxy) phenyl]-porphyrin and its tetramethylated derivative 5,10,15,20-tetrakis [4-(3-*N*,*N*,*N*-trimethylammoniumpropoxy) phenyl]porphyrin tetraiodide [198]. It turned out that the initial charge of the molecule had no effect in vitro on *C. albicans* inactivation because the amino groups were protonated in physiological medium conditions. The important observation was that PACT combined with fluconazole chemotherapy potentiated the antifungal effect of both therapies [198]. Cationic porphyrins were also used in the study by Gomes et al. [199], who assessed the effectiveness towards photoinactivation of *Penicillium chrysogenum* conidia by two series of porphyrins, based on the scaffold of either 5,10,15,20-tetrakis (pyrid−4-yl) porphyrin or 5,10,15,20-tetrakis [2,3,5,6-tetrafluoro−4-(pyrid−4-ylsulfanyl) phenyl]porphyrin, substituted on nitrogens with various alkyl substituents. It was found that although all of the PSs exhibited efficient singlet oxygen generation yields, only **17** and 5,10,15,20-tetrakis (*N*-pentylpyrid−4-yl) porphyrin tetraiodide were able to reduce the viability of the fungal cells, inducing 4.1 and 3.4 log reduction, respectively. Authors concluded that increasing the length of the alkyl substituent increased the affinity of the PS to the fungal cell, while at the same time decreasing the water-solubility and causing increased aggregation [199]. Moreover, **17** was tested not only in vitro but also in vivo by Mitra et al. [200]. First, the effectiveness in photoinactivation of *C*. *albicans* was assessed and compared to results obtained for PACT with **1** as a PS. It was found that use of **17** yielded over 3 log reduction more than **1** when the same concentrations were used. When applied to a mouse model of *Candida* infection, the porphyrin was found to selectively accumulate in fungal cells. The selection of appropriate formulation was shown to have a significant effect on the photokilling efficacy, as ethanol/glycerol/water was found superior to glycerol/water medium (~1.5 versus <1.0 log reduction, respectively). Importantly, although the treated tissue showed some changes after the PACT, it was completely restored over nineteen days [200]. The mode of action of **17** on *C*. *albicans* was well established [201]. It was demonstrated that it in fact binds strongly to the cell membrane and is not internalized, and the influx of the PS occurs only after cell death. The permeability increase was observed when additional osmotic stress was applied by culturing the fungi in hypoosmotic medium. In another study, it was noticed in confocal fluorescence spectroscopy that the tetratosylate derivative of this PS dissolved in pure water and added to the culture was distributed throughout the *C. albicans* cell upon 10 min incubation, both in yeast and filamentous forms [200]. Apart from that, Quiroga et al. made an important observation—when *C. albicans* immobilized cells were treated with **17**, the amount of the PS decreased as the cells were washed with PBS [190]. The effect of number of cationic charges in the PS molecule was also studied, when Gonzales et al. compared **17** and 5,15-bis [4- (3-trimethylammoniumpropyloxy) phenyl]porphyrin dichloride (**22**, Figure 7) as PSs for PACT against *C*. *albicans* biofilms and planktonic cultures [202]. It turned out that the dicationic porphyrin induced a reduction of over 6 log in planktonic *C*. *albicans*, which was similar to the activity of **17** used at four times higher concentration, and a 5 log reduction in the fungal biofilm, greatly surpassing the effectiveness of the tetracationic porphyrin, as 50-times the concentration of the tetracationic porphyrin yielded the same effect. Probably, the presence of four charges results in stronger interaction with the cells and prevents the porphyrin from diffusion into the biofilm as it is bound on its surface [202]. An alternative approach was presented by Vandresen et al. who assessed the effectiveness of a series of porphyrins substituted in *meso* positions with (*N*-methyl) pyrid−4-yl and phenyl rings in different ratios in PACT of *Colletotrichum graminicola* [203]. The results showed that the A_3_B derivative, namely 5,10,15-tris (*N*-methylpyrid−4-yl) −20-phenylporphyrin triiodide, exhibited the best activity in PACT, which authors associated with the amphiphilicity of those molecules. Surprisingly, AABB derivative was found to exert strong antifungal activity per se, even without the irradiation [203].

Smijs and Schuitmaker screened a series of PSs for their potential use in photoinactivation of *T. rubrum* [204]. The tested compounds included deuterioporphyrin and its monomethyl ester, 5,10,15-tris (*N*-methylpiryd−4-yl) −20-phenylporphyrin trichloride (Sylsens B, **23**, Figure 7), hematoporphyrin, porfimer sodium (from Photofrin^®^), zinc (II) phthalocyanine, phthalocyanine tetrasulfonic acid, and chloroaluminium (III) phthalocyanine tetrasulfonic acid. It was found that porfimer sodium and phthalocyanines were less active in PACT experiments, exhibiting a fungistatic effect, while the tested porphyrins induced photokilling of the fungus. Among them **23** and hematoporphyrin monomethyl ester were the most effective PSs but Sylsens B exhibited no dark toxicity [204]. The topic was continued, as Smijs et al. reported assessment of the PACT of those two porphyrins on *T. rubrum* grown on human stratum corneum ex vivo model [205]. The authors noticed that the efficacy of PACT was strongly dependent on the time after the inoculation of the fungus, as well as the medium used for application of the PS [205]. Further research was focused on the optimal conditions for PACT using **23** and deuterioporphyrin monomethyl ester (**24**, Figure 7) [206]. These studies revealed that **23** was a better PS when such optimal conditions of pH~5 and low ionic strength of the medium were applied [206]. This acquired information was used in a following study aimed to determine the usefulness of **23** towards photoinactivation of reference *T. rubrum* and its clinical isolate [207]. Different modes of application and dosimetry were tested for efficient photokilling of the fungi. The authors also highlighted the possibility of penetration of **23** to the skin when it was applied at pH 7.4 but not at pH 5.2. However, use of a surfactant made it possible for the PS to cross the stratum corneum barrier [207]. Further studies were performed to test the efficacy of PACT of **23** and its analogues—5,10,15-tris (*N*-methylpyrid−4-yl)−20- (4-sulfanylphenyl)porphyrin trichloride and 5,10,15-tris(*N*-methylpyrid−4-yl)−20-{4-[(1-methoxy−1-oxo−3-sulfanylpropan−2-yl)amino]−4-oxobutoxyphenyl}porphyrin trichloride (**25**, Figure 7) [208]. The PSs were assessed in in vitro photoinactivation of a clinical isolate of *T. mentagrophytes*, as well as ex vivo permeation into the nail. *T*. *mentagrophytes* viability was decreased upon irradiation with the PSs, 4.6, 4.4 and 3.2 log reduction was recorded for 5,10,15-tris (*N*-methylpyrid−4-yl)−20- (4-sulfanylphenyl)porphyrin trichloride, **23** and **25**, respectively, when the same conditions were applied. However, the latter was more photostable and more active when low oxygen concentration conditions were assessed [208]. This research was continued by Hollander et al., who tested the effectiveness of using **25** as a PS in PACT against ex vivo onychomycosis caused by either *T. rubrum*, *T. mentagrophytes*, or *Trichophyton tonsurans* [209]. Each of the microorganisms was affected by the photodynamic treatment, as the viabilities decreased in every case at least by 70% after single irradiation. After the second irradiation all the onychomycoses were cured. The authors report that the effectiveness was compared to another PS, also bearing three cationic charges—**23**, and was proven superior suggesting that the free thiol group played an important role in the internalization of the macrocycle. Moreover, although both porphyrins were structurally similar, **23** acted mainly via II type of photodynamic reaction (i.e., singlet oxygen production) and the studied **25**—via type I of photodynamic reaction [209].

### 6.3. Porphyrin Containing Materials

Alvarez et al. prepared thin films of poly (silesesquioxane) in which 5-(4-carboxyphenyl)-10,15,20-tris (4-methylphenyl)porphine (**26**, Figure 7) was embedded [210]. Physicochemical and photochemical characterization of the films revealed their promising photosensitizing properties and the ability to generate singlet oxygen, as assessed using 9,10-dimethylanthracene and 9,10-anthracenediylbis (methylene)dimalonic acid. The material was then tested for its photodynamic properties by photooxidation of tryptophan. Further in vitro experiments against *C. albicans* showed that the irradiation of the prepared films induce near 2.5 log reduction. Further studies revealed that this immobilized porphyrin acts using mostly the II type photodynamic reaction [210]. Viana et al. prepared a material based on CdTe quantum dots combined with Zn (II) 5,10,15,20-tetrakis (*N*-ethylpyrid-2-yl) porphyrin [211]. The material was then characterized and assessed by means of its photochemical properties showing ability to generate ROS. The application of the material to the PACT against *C. albicans* showed that it is a much less potent PS than the parent porphyrin alone (1 and 3 log reductions, respectively). Such effect was attributed to strong connection between the porphyrin and quantum dot, which did not enable the internalization of PS into the cells [211]. A successful nanomaterial for fighting *T. rubrum* was developed by Wijesiri et al., who combined silver core-silica shell nanoparticles with hematoporphyrin IX [212]. This material applied to *T. rubrum* induced a 3 log reduction while for each of its components this value did not exceed 1 [212]. The activity of porphyrins is summarized in Table 4.

## 7. Chlorins

**Figure 8:**
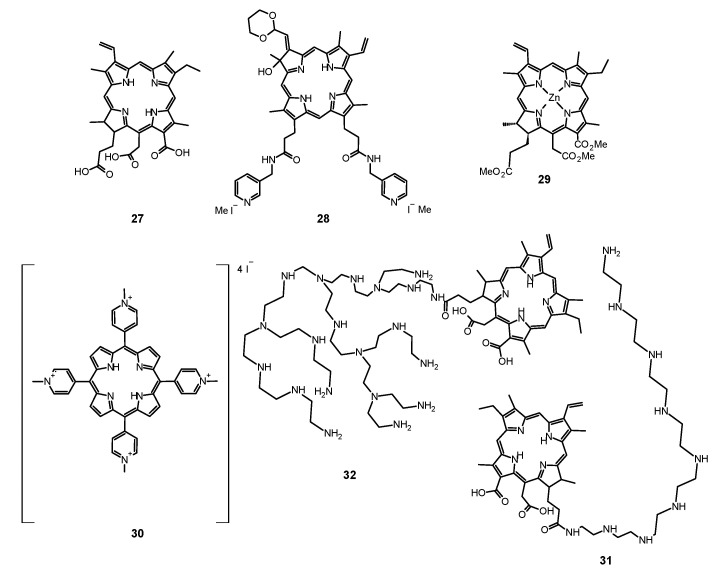
Structures of chlorins (**27**–**32**).

Chlorins are made up of four pyrrole rings with reduced bond in one of them. The most popular chlorin is chlorin e6 (**27**, Figure 8). It is the second generation of PSs and can be excited with a 660–670 nm light. Moreover, **27** is characterized by easy synthesis and high quantum yield of singlet oxygen after irradiation as well as low toxicity in the dark. However, its disadvantage is low solubility in water. Therefore, the modification of the molecule or the use with drug carriers ensuring enhanced antimicrobial photoactivity are needed [19,214].

**Figure 9:**
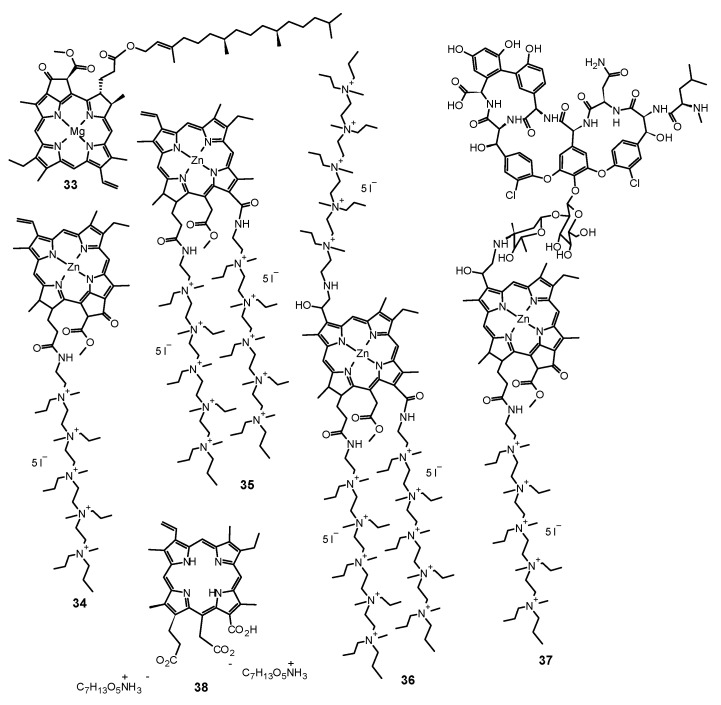
Chemical structures of chlorins (**33**–**38**).

Sueoka et al. investigated cationic chlorin derivative TONS 504 (**28**, Figure 8) excited with LED (660 nm) against *Fusarium solani* and *Aspergillus fumigatus* [215]. Concentration of 1 mg/L and LED irradiation at the dose of 30 J/cm^2^ or 10 mg/L and light dose of 10 J/cm^2^ was enough for complete elimination of *F. solani*, whereas *A. fumigatus* was more resistant. It was possible to achieve a significant but not complete reduction but a higher concentration and a light dose (10 mg/L; 30 J/cm^2^) were required [215]. Carvalho et al. observed biofilm formation inhibition using **27** [216]. Biofilm growth was reduced about 90% using 20 μM PS and LED light (660 nm) at the dose of 40 J/cm^2^. Its effectiveness is due to an increasement of the cell membrane permeability after PACT, about 5 times compared to control [216]. Diogo et al. compared the PACT activity of Zn (II) chlorin e6 methyl ester (**29**, Figure 8) and other PSs (**3**, **10**, tetracationic porphyrin—**30**) as well as conventional agents (3% NaOCl, 17% EDTA and 2% chlorhexidine gluconate—CHX) against *E. faecalis* and *C. albicans* mono and multispecies biofilms. **27** and tetracationic porphyrin activated with light showed the highest reduction rate in biofilm among PSs studied. Noticed activity was similar to that obtained for CHX and EDTA; **29** coupled with red LED light (627 nm) removed 58.98% of dual-species biofilm. NaOCl showed the highest antimicrobial effectiveness, but its disadvantage is high cytotoxicity [217]. In order to improve **27** photodynamic activity, Yang et al. encapsuled the PS in positive charged liposomes composed of cetyltrimethyl ammonium bromide (CTAB) and dimyristoyl-*sn*-glycero-phosphatidylcholine [218]. Encapsulation prevents aggregation of PS, which leads to the decreasing of singlet oxygen formation. Moreover, the positive charges increase the affinity for the negatively charged wall of *C. albicans*. Increasing the ratio of CTAB to lipids leads to an increase in the zeta potential and increases binding to the yeast. At a low lipid:CTAB ratio (10:1), no significant difference in inactivation was noticed between not bound **27** and that placed in liposomes. However, total elimination of *C. albicans* was achieved with a lipid:CTAB ratio of 10:3 and **27** a concentration of 10 µM. The higher CTAB amount resulted in increase of reductions of *C. albicans* growth (up to 6 log). It was pointed out that the efficacy of PACT is also dependent on particle size and PS concentration. It was found that PS loaded into smaller liposomes, (80 nm in comparison to 117 nm), showed much higher photodynamic antimicrobial activity. The use of liposomes with positively charged CTAB particles also allowed to increase in vivo reduction rate from 1.00 log to 1.75 log when compared with free-from and liposomes without CTAB in Rat Skin Burn Wound [218]. Tegos et al. have studied another approach to increase the efficacy of **27** [219]. They investigated the influence of conjugation of polyethyleneimine (PEI) and **27** on the activity against several pathogenic microbes including: Gram-negative bacteria—*E. coli* and *P. aeruginosa* and Gram-positive bacteria—*S. aureus* and *S. pyogenes* as well as yeast—*C. albicans*. The authors suggest that the advantage of PEI conjugates is the lack of peptide bonds susceptible to degradation by protease enzymes. Moreover, **27** conjugates were coupled with three different PEI preparations: linear PEI (**31**, Figure 8), low molecular weight [LMW] (**32**, Figure 8), high molecular weight [HMW]. Against Gram-positive bacteria, the concentration of 1 µM of each conjugate was used, while against Gram-negative and *C. albicans*, the concentration was increased to 10 µM, and the light dose did not exceed 100 J/cm^2^. Furthermore, **32** proved to have the least photodynamic antimicrobial activity against any of the species, while HMW proved to be the most active. A reasonable reduction in bacterial growth was achieved. Interestingly, they noticed that *C. albicans* elimination by PACT treatment with HMW and linear PEI was the most effective (4 to 6 logs reduction with 16 J/cm^2^). Pure **27** revealed 2.5 logs of *C. albicans* reduction at the light dose of 40 J/cm^2^ while the LMW gave only 2 logs at the light dose of 100 J/cm^2^. Authors reported that all PSs tested produced the same amount of superoxide, whereas the most efficient singlet oxygen generator was **27** before **32**. Interestingly, photodynamically most active conjugates (**31**, and HMW) were the worst ^1^O_2_ producers. It was suggested that this phenomenon may be caused by the lack of an appropriate tertiary structure of **31** and **32** conjugates [219]. Not only **27** is extensively studied as a PS against yeast, but there are other interesting compounds based on the chlorin macrocyclic ring. Astuty et al. investigated the potential of chlorophyll extract of papaya leaf as PS (i.e., chlorophyll a—**33**, Figure 9) [220]. Although, it was reported that obtained extract revealed some activity in the *C. albicans* biofilm combat (reduction up to 32%) in vivo experiments or potential clinical use of this PSs may be problematic. The extract presented is a mixture of PSs of unknown composition. This makes it nearly impossible to propose proper dosimetry (PS doses as well as light doses) to achieve the intended therapeutic effect. Huang et al. obtained cationic pheophytin bearing 5 (**34**, Figure 9), 10 (**35**, Figure 9), or 15 (**36**, Figure 9) quaternary ammonium groups [221].

Moreover, they developed one conjugate of cationic PS possessing five charged centers with vancomycin (Van) molecule (**37**, Figure 9). Van is an antibiotic, which inhibits cell wall synthesis. It was proved that all studied conjugates are able to singlet oxygen formation; thus, authors claimed that they work according to the II type of photodynamic action. Interestingly, the presence of more than one pentacationic chain in the conjugate structure increased molecular dispersion. It reduced aggregation and in consequence increased singlet oxygen production yield. In the photodynamic antimicrobial activity assessment, two light sources were used: the blue LED (415 ± 15 nm) and red (660 ± 15 nm) lights. Compound **34** at the concentration 100 µM excited with blue light at the dose of 20 J/cm^2^ eradicated the studied bacteria. Remarkably, a derivative bearing ten positive charges of **35** needed a higher concentration (500 µM) and a light dose of 20 J/cm^2^ to obtain the same effect. Unfortunately, excitation of PSs with red light led to much lower bactericidal effect. Authors claimed that it results from low absorption molar coefficient for these wavelengths, and thus, a lower amount of photons (energy) could be absorbed. In the case of experiments on combating *C. albicans*, they noticed that for total eradication, 100 µM of **34** (20 J/cm^2^) is needed. Interestingly, the same effect was provided by **35** in concentration of 10 µM (20 J/cm^2^). Noteworthy is the fact that a conjugate of PS with Van was found completely inactive against yeast. The most effective against *Candida* was conjugate **36**, which for eradication needs to be used in a concentration of 50 µM and a light dose of 10 J/cm^2^ [221]. It clearly indicates that the important factor for photodynamic *Candida* combat is the amount of positive charges in the structure of PS. An approach in which the excitation factor in PDT (the light) is being replaced by ultrasounds is becoming more and more popular opening up new method sonodynamic therapy (SDT). This modification enables to reach targets (microbes, cancer cells) located deeply inside the tissue. Alves et al. have tested sono- and photodynamic activity against *C. albicans* of photodithazine^®^ (**38**, Figure 9) [222]. It was noticed that ultrasounds at the power of 2.5 W/cm^2^ mediated **38** at the concentration of 50 mg/L resulted in total elimination of the yeast (25 mg/L resulted in 4.35 log of growth reduction). Interestingly, PS at the same concentration was excited with LED light at the dose of 25 J/cm^2^ giving 5.87 log reduction. In biofilm form, *C. albicans* revealed high resistance against PACT and SDT applied separately. Totally different observations were made when both modalities were combined. In this experiment authors used excitation sources simultaneously with the same parameters described above. This caused a significant reduction in *C. albicans* biofilm growth, about 3.39 log for PS concentration equal 200 mg/L [222]. The reported facts need further studies. Combination of chlorin with conventional antifungals has been studied as well. In one of the experiments, nystatin was included in the standard treatment with **38** [223]. This study was performed on mice infected with fluconazole-resistant *C. albicans*. The use of antifungal therapy combined with PACT not only increased its effectiveness (reduction of up to 2.6 logs) but also increased the expression of p21 and p53 proteins. There was also a marked reduction in inflammation and pathological changes in the tongues and mouths of infected rodents. Interestingly, the best results were achieved when PACT was used first and nystatin afterwards. Only 2.1 log reduction was obtained with the reversed order. Animals treated with PACT alone had a 1.3 log reduction in fungal cell counts, while nystatin alone had an effect of 1.1 log reduction, which shows a synergy between the therapies [223]. The activity of chlorins is summarized in Table 5.

## 8. Porphyrazines and Phthalocyanines

**Figure 10:**
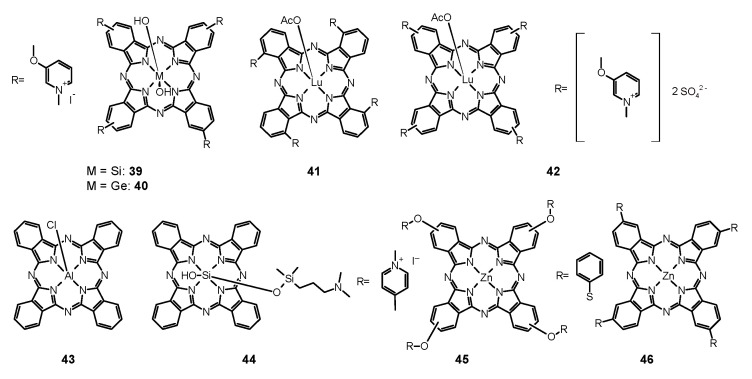
Chemical structures of (**39**–**46**).

Phthalocyanines (Pcs) are molecules similar to porphyrins but with a larger macrocycle ring, and they absorb longer wavelengths of light more intensively [13,227,228]. Scientists also compared the effectiveness of PACT mediated with pcs against planktonic and biofilm forms of pathogens. Hsieh et al. performed research on the use of PACT to target *C. tropicalis* and to improve the cellular penetration of the tested pc PS [229]. They prepared cationic chitosan/tripolyphosphate NPs to encapsulate pc which enhanced its uptake four-fold. PACT effectively eradicated 80% of planktonic *C. tropicalis*. However, to eliminate adherent *C. tropicalis*, sequential treatment with PACT and flucytosine had to be implemented to eradicate pseudohyphae and yeast-like *C. tropicalis* cells [229]. Mantareva et al. synthesized water-soluble pc complexes of silicon (**39**, Figure 10) and germanium (**40**, Figure 10) to test their photodynamic efficacy against *C. albicans* both in planktonic and biofilm form [230]. In water medium, the **39** was slightly aggregated, whereas **40** revealed a strong aggregation effect. After **39** (1.8 mM) mediated PACT upon soft light radiation (50 J/cm^2^), pathogenic cells in suspension were inactivated, contrary to the negligible effects after strong treatment conditions with **40**. Only after application of fractionated LEDs radiation (three times per 50 J/cm^2^), mature biofilm of *C. albicans* grown on denture was eliminated. It turned out that a water-soluble **39** can be considered as a potential PS in PACT against *C. albicans* [230]. The same authors have continued their studies focused on PACT method [231]. In 2016, they synthesized water-soluble cationic lutetium (III) acetate Pcs (**41**,**42**, Figure 10) as potential PSs in PACT towards the fungus *C. albicans* and the Gram-negative bacteria—*P. aeruginosa* both in form of suspensions and biofilms. The levels of their uptakes were higher for fungal cells than for bacterial cells, and 100% penetration of PSs into fungal biofilms was observed. Gentle irradiation with LED at 665 nm (50 J/cm^2^) and 20 μM **41** applied against bacteria or 30 μM **41** against fungi appeared to be sufficient for the complete photoinactivation of pathogens in planktonic cultures. However, the PACT towards both species tested in form of biofilms was not satisfactory [231]. Ribeiro et al. studied the usefulness of new drug delivery systems for PACT against *C. albicans* both in planktonic cultures and biofilm [232]. The scientists developed cationic and anionic nanoemulsions (NE) for entrapment of hydrophobic aluminum(III) phthalocyanine chloride (**43**, Figure 10). Various factors such as the delivery system, the charge, and the light dose influenced the viability of *C. albicans*. Cationic NE-**43** lowered substantially both colony counts and cell metabolism. It also led to significant destruction of the cell membrane. The illumination with higher light dose (100 J/cm^2^ instead of 50 J/cm^2^) did not result in greater reduction of *C. albicans* metabolism, differently from the colony count assay. In the case of the biofilms, it reduced cell metabolism by 70%. Interestingly, anionic NE-**43** did not reveal antifungal potential [232]. Morgado et al. proposed **43** mediated PACT as an alternative to the long lasting and causing side effects of the conventional treatment of onychomycosis [233]. They studied key properties of PACT, with the PS placed in nanoemulsions, as a a drug carrier in a proof-of-concept clinical trial. The clinical protocol turned out to be safe, and its antifungal effectiveness could be comparable to that of conventional treatments. Of treated lesions, 60% were cured, and no adverse effects, thanks to the local therapy, were observed. Moreover, the possibility of developing fungal resistance was ruled out [233]. Rodrigues et al. studied the photodynamic effects of the water soluble PS **43** entrapped in nanoemulsion (**43**/NE) on the in vitro melanized cells of *Cryptococcus neoformans* [234]. Elimination of cells depended on a PS concentration and a light dose. Treatment with concentrations of 4.5 μM **43**/NE and a light dose of 10 J/cm^2^ caused a significant decrease in survival (reduction up to 6 logs). The degree of **43** uptake by *C. neoformans* melanized cells rose with the increase of the PS concentration. Internalization of **43** by *C. neoformans* was confirmed by confocal fluorescence microscopy. Such kind of PACT was considered as an alternative treatment option for fungal localized lesions [234]. The scientists examined further the in vitro sensitivity of *C. albicans* and *C. tropicalis* to PACT with **43**/NE [235]. They compared the cell survival before and after washing out the not bound PS. Light exposure without the PS and treatment with the PS only did not eliminate the pathogens. PACT with **43**/NE decreased by five orders of magnitude viability for *C. albicans* and between four and five orders of magnitude for *C. tropicalis*. Washing the cells before irradiation did not reduce fungal inactivation, which indicated cell bound **43** as the main photosensitization promoters. Uptake of the PS depended on its concentration. Internalization of **43** by both fungal pathogens was confirmed by confocal fluorescence microscopy. PS appeared to accumulate in certain areas of cytoplasm. Aluminum(III) phthalocyanine chloride nanoemulsion can be considered as an promising formulation applied for PACT to eliminate both *C. albicans* and *C. tropicalis* [235]. Lam et al. focused on Pc 4 (**44**, Figure 10) mediated PACT only in planktonic *C. albicans,* and they evaluated the in vitro cytotoxicity and mechanism of the treatment [236]. Using confocal microscopy, they confirmed localization of **44** in cytosolic organelles, including mitochondria. In a colony formation assay, they observed a reduction of pathogenic cell survival by 4 logs at 1.0 μM of **44** and light irradiation at 2.0 J/cm^2^. This kind of treatment led to impaired fungal metabolic activity and changes in nuclear morphology typical of apoptosis [236]. The scientists also evaluated the antifungal potential of metalated pcs to optimize the formulations containing the PS and the dosimetry. Carmello et al. compared the effects of photoinactivation of *C. albicans* in a murine model of oral candidiasis using as PS: **43** encapsulated in cationic nanoemulsions (NE) and **43** diluted in DMSO. After induction of oral candidiasis in mice, PACT was applied to the tongue, with LED light (100 J/cm^2^, at a wavelength of 660 nm). After twenty-four hours and 7 days, *C. albicans* were recovered from the tongue of animals which were sacrificed, and their tongues were analyzed. **43**-NE-mediated PACT eliminated 2.26 log of *C. albicans* in comparison to the control group. It turned out that PDT mediated by chloro-aluminum phthalocyanine entrapped in cationic NE was effective in eliminating *C. albicans* in the mural oral lesions in contrary to animals treated with PDT mediated by **43** formulated in DMSO, where no decrease in cell viability of fungal pathogen was observed [237]. Di Palma et al., investigated cellular uptake and photodynamic action of zinc (II) 2,9,16,23-tetrakis-4- (*N*-methylpyridyloxy) phthalocyanine tetraiodide (**45**, Figure 10) in *C. albicans* [238].

**Figure 11:**
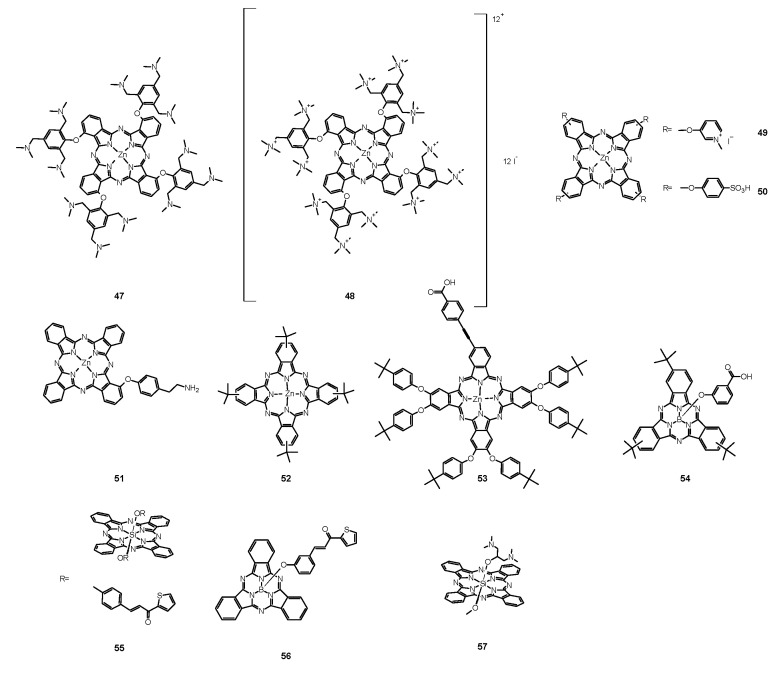
Chemical structures of (**47**–**57**).

In in vitro studies, they observed that **45** was rapidly bound to the fungal cells and the binding process depended on **45** concentrations and cell densities. With the 10-fold reduction of the cell density, 10-fold increase in cell uptake of **45** was observed. A significant amount of **45** remaining in the cells even after two washing steps indicated an affinity-mediated binding mechanism between the PS and *C. albicans*. Moreover, **45** turned out to be a promising PS for photodynamic inactivation of the pathogen [238]. Di Palma et al. continued their research on the PACT of *C. albicans* cells mediated by **45** [239]. They studied photodynamic mechanism and the photoreaction type I/II pathways mediated by this PS. They concluded that both photoprocesses can happen simultaneously, and the ratio between the reactions can be influenced by the nature of the PS, its location in the various areas of microbial cells, the PACT treatment, and the kind of pathogen cells. An oxygen atmosphere was required for an efficient photokilling and photoinactivation of fungi. Moreover, **45** can be considered as a PS predominantly acting through the intermediacy of O_2_. However, a few other ROS can be involved in the PACT as well [239]. Junqueira et al. assessed the effectiveness of photodynamic treatment of fungal infections and investigated the use of a cationic NE of zinc 2,9,16,23 tetrakis (phenylthio)−29*H*,31*H*-phthalocyanine (**46**, Figure 10) as a component of PACT against biofilms formed by *Candida* spp., *Trichosporon mucoides*, and *Kodamaea ohmeri* [240]. After treatment of pathogenic biofilms with the PS and a gallium-aluminum-arsenide GaAlAs laser (26.3 J/cm^2^), the microbiological examination of biofilm cells was performed to estimate the number of CFUs. Although all biofilms studied were susceptible to PACT, the strains of *Candida* genus appeared more resistant to PACT than emerging pathogens *T. mucoides* and *K. ohmeri*. Therefore, a procedure combining nanostructured formulations of cationic **46** with an irradiation with a laser seems useful to reduce the number of cells in the biofilms formed by the opportunistic yeasts studied [240]. Lam et al. studied the possibility of the application of silicon **44** mediated PACT against very difficult to cure infections caused by *T. rubrum* [241]. Traditional therapy usually involves drugs such as terbinafine, ciclopirox, or itraconazole, causing severe side effects, including liver toxicity. To improve topical treatment results, application of a noninvasive PACT was considered. The scientists tested the cytotoxicity of PACT in vitro using the **44** against *T. rubrum*. They analyzed confocal microscopy images and observed that **44** bound to cytoplasmic organelles, and ROS was generated upon irradiation. They found out that PACT consisting of 1 μM of **44** combined with irradiation with light at 2.0 J/cm^2^ at the wavelength of 670–675 nm was effective in reducing the overall cell survival rate of *T. rubrum*, both terbinafine-sensitive and terbinafine-resistant strains [241]. Derivatives of different charges of phthalocyanine were studied by Li et al. in terms of their potential application of a new tetra-α-substituted zinc(II) phthalocyanine as a PS for PACT against *C. albicans* [242]. They examined two derivatives-containing Pc with twelve amino groups (**47**, Figure 11) and their fully quaternized analogue (**48**, Figure 11). The dodeca-cationic phthalocyanine revealed a higher photodynamic inactivation potential against *C. albicans* due to its non-aggregated form in aqueous media and efficient cellular uptake. Moreover, it exhibited stronger affinity to *C. albicans* cells than mammalian cells. These properties made it a promising selective antifungal PS [242]. Mantareva et al. synthesized the cationic zinc(II) 2,9,16,23-tetrakis (*N*-methylpyrid−3-yloxy)phthalocyanine (**49**, Figure 11) and the anionic zinc(II) 2,9,16,23-tetrakis(4-sulfophenoxy) phthalocyanine, (**50**, Figure 11) [243]. They investigated their photodynamic activity towards three pathogenic microorganisms—the Gram-positive bacteria *S. aureus*, the Gram-negative *P. aeruginosa*, and the fungi *C. albicans.* Greater drug uptake was observed in case of lower cell density. Cell density influenced also the binding potential of the PS before irradiation. The cationic PS completely inactivated *S. aureus* and *C. albicans*, but with 4 log eliminated the Gram-negative *P. aeruginosa*, which was able to accumulate two order lower amounts of the studied PSs in comparison to other pathogens. Tested *S. aureus* exhibited complete inactivation after mild photoinactivation treatment with the **49** (1.5 µM, 30 J/cm^2^). For the effective treatment of *P. aeruginosa* and *C. albicans* with **49,** higher concentration and light doses were required. Similarly, the high drug concentration (6 μM) of the **50** combined with irradiation of light (60 J/cm^2^) was necessary to combat both the fungi and bacteria species studied. Therefore, the **49** turned out to better promote photodynamic inactivation of pathogens than the anionic one [243]. Tang et al. prepared four conjugates with various contents of pc, and their derivatives with different quaternization degrees to test their antifungal acitivity on *C. albicans* [244]. To improve the water solubility of the PS and therefore the therapeutic efficacy of PACT, two carboxymethyl chitosans (CMC) of the molecular weight 1.50 kDa and 170 kDa were conjugated with zinc(II) 1-[(aminoeth−2-yl) phen−4-yloxy] phthalocyanine (**51**, Figure 11) and further quaternized to obtain eight novel conjugates. All conjugates showed the improved water solubility of **51** and its decreased aggregation rate in water. The lower-molecular-weight CMC-conjugated **51** turned out to be more easily ingested and highly photoactive because of its greatest uptake by fungal cells. This conjugate exhibited the highest photocytotoxicity with an IC_90_ value down to 0.72 μM, whereas further quaternization led to the reduction of the photodynamic antifungal activity. Interestingly, both the unquaternized and quaternized conjugates revealed special affinity to the mitochondria of *C. albicans* [244]. Ozturk et al. studied the antifungal photodynamic potential of five Pc derivatives: **52**, **53**, **54**, **55,** and **56** and materials **53**-TiO_2_, **54**-TiO_2_ towards *C. albicans* [245]. They evaluated the minimum inhibitory concentration (MIC), uptake of the compounds in fungal pathogen and dark toxicity. Application of PS at concentration of 64 μg/mL resulted in maximum uptake of each compound into the cells with the exception of **53**. PSs did not reveal dark toxicity effects at sub-MIC concentrations in comparison to the negative control groups. **52**, **53**, and **53**-TiO_2_ exhibited inhibitory action after irradiation with the light dose of 90 J/cm^2^ in the presence of the compounds. In addition to the fungicidal effects, **54**, **54**-TiO_2_, **55**, and **56** compounds inhibited the growth of *C. albicans* after irradiation [245]. Another Pc derivative, an axially-substituted unsymmetrical bisamino phthalocyanine (**57**, Figure 11) was investigated by So et al. in terms of its antifungal effect on *C. albicans* depending on the dose [246]. The cell viability of the fungus was determined by the colony formation assay. It decreased to about 10% at 0.02 μM **57** excited with light at the dose of 12 J/cm^2^. After a short incubation time of 5–15 min, the PS was able to achieve its optimal antifungal potential. Due to confocal scanning laser microscopy, the scientists observed effective internalization of **57** by the fungal cells. The investigated PACT approach resulted in the intracellular ROS production and affected the membrane integrity of the pathogen cells which had been tested with the high affinity acid stained with propidium iodide. In case of intact membranes, PI should not penetrate through to interact with DNA. However, in this study, an increased number of high fluorescence intensity cells was found out in the flow cytometry, which reflected higher membrane permeability after photodynamic reaction. Results depended on the dose of the PS applied and correlated both with cell viability and ROS generation [246]. The activity of porphyrazines and phthalocyanines is summarized in Table 6.

## 9. Other Photosensitizers

**Figure 12:**
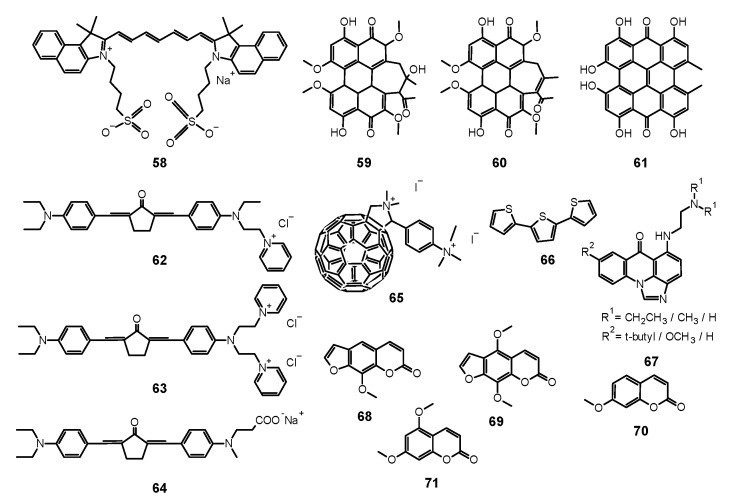
Chemical structures of **58**–**71**.

An interesting candidate for PACT is indocyanine green (**58**, Figure 12), PS accepted by FDA to diagnostic procedures. Therefore, an advantage of this agent is proven safety in clinical use. In Europe, it is manufactured in commercial preparation Emundo^®^ (A.R.C laser GmbH, Nurnberg, Germany) as a PS for photodynamic (PDT) and photothermal therapy (PTT) of infections followed inflammations in dentistry (i.e., periimplantitis) [247]. In addition, **58** can absorb light at the wavelengths of 810 ± 10 nm. After photon absorption it is excited. Further, the molecule is deactivated by heat emission. This causes destruction of cells in the neighborhood of PS. On the other hand, **58** excited with lower wavelengths works as a typical PS in PDT. Fekrazad et al. reported activity of **58** in decreasing growth rate of *C. albicans* and *T. rubrum* [74,85,248]. Nevertheless, the reduction was found out at the level of ca. 1 log [74,79,85,248], simultaneously Afroozi et al. indicated remarkable adjuvant impact of ICG on the conventional treatment of denture stomatitis caused by *C. albicans* infection with nystatin [249]. It should be mentioned that Azizi et al. reported the same growth inhibition of *C. albicans* by PACT mediated **58** and conventional antiseptic agents like chlorhexidine and the antifungal drug nystatin [79].

Another interesting PS worth mentioning is hypocrellin A (**59**, Figure 12) and B (**60**, Figure 12). These compounds are perylenequinone dyes derived from *Hypocrella bambusae* [250]. Yang et al. studied **59** as a candidate for a PS for *C. albicans* combat [251]. Furthermore, **59** is known as an efficient producer of ROS including singlet oxygen, hydroxyl and superoxide radicals [5]. Yang et al. tested **59** in yeast eradication potential as well as in in vitro and in vivo. In vitro experiments indicated that **59** excited with white light caused reasonable decreasing of *C. albicans* growth. Moreover, after PACT procedure disruption of the membrane integrity was noticed, which enabled PS to enter the cell, **59** reached endoplasmic reticulum as well as Golgi bodies. In-depth analysis of the after-effects of PACT-**59** indicated that metacaspase activation, DNA fragmentation, and nuclear condensation appeared in the cell. This led to cell death on the apoptosis pathway. In the in vivo studies on the murine model, an intensified PACT effect was observed. In the wound infection area, a decrease in the CFU of yeast was noted. Authors explained such phenomenon by immune response reported before activation [251]. Jan et al. evaluated **60** as a PS against yeast. They reported that **60** irradiated with white light enables growth reduction up to 4.6 log (10 µM) and even 7.0 log (100 µM) for azole-sensitive and azole-resistant *C. albicans* strains [252].

Hypericin (**61**, Figure 12) also was evaluated as a PS in PACT against fungi. Chemically **61** is a naphthodianthrone derivative isolated from *Hypericum perforatum* [253]. Rezusta et al. assessed the ability of **61** in *Candida* species eradication [254]. It was noticed that for high reduction at the level of 3 log dependent on species, different concentrations of PS irradiated with light at wavelength of 602 ± 10 nm at the dose of 18 J/cm^2^ are needed. Moreover, **61** should be used at 0.625–1.250 µM against *C. albicans*, 2.5 µM against *C. parapsilosis*, and 40 µM against *C. krusei*. Interestingly, compared to the other PSs, for high activity, a short incubation time was needed (below 1 min). Increase of incubation time did not result in increase of its efficacy. The authors tested also the toxicity of **61** at the same dosimetry against human keratinocytes and observed no negative impact of PACT protocol [254]. Rezusta et al. continued their studies and performed evaluation of usability of PACT-**61** in dermatophytes combat—*T. rubrum* and *T. mentagrophytes* [255]. To achieve the 3 log reduction level against the mentioned fungi, PS needed to be excited with light (602 ± 10 nm) at the dose of 37 J/cm^2^. It was assessed that against *T. rubrum* **61** concentrations in the range of 10–20 µM are necessary and against *T. mentagrophytes* in the range of 20–50 µM. Additionally, it was observed that **61** is well spread in the conidia and hyphae cytoplasm [255].

Unusual structures of PSs were proposed by Zhou et al. for combat with *C. albicans* fluconazole-resistant strains [256]. The authors indicated that benzylidene cyclopentanone PSs: **62**, **63** and **64** reveal high photodynamic potential against resistant yeast strains. The strain of *C. albicans cal-1* after excitation with light at the wavelength of 532 nm and dose of 24 J/cm^2^ was reduced about 3.76 log by **62** (7.5 µM), about 3.08 log by **63** (7.5 µM), and about 3.72 log by **64** (25 µM). It was observed that total fungi eradication was achieved for **62** and **63** at the concentration of 25 µM. The detailed studies enabled to depict the depth of after-effects inside the fungal cell after turning on photodynamic process. It was discovered that **63** works only outside the cell and impacts the wall functions. PS **62** was located inside the cell in the whole volume of cytoplasm and destructed its structures. Interestingly, **64** migrated into the cells slightly disrupting cell membrane. Finally, **64** reached mitochondria and efficiently destroyed them [256].

It is well known that fullerene and its derivatives are able to form ROS [257]. Therefore, Milanesio et al. assessed the efficacy of fullerene derivative (**65**, Figure 12) photodynamic inactivation of *C. albicans* [258]. PS (10 µM) activated with white light at the dose of 162 J/cm^2^ revealed extremely high reduction of yeast growth ca. 5 log [258].

An initial experiment of photodynamic activity of α-terthienyl (**66**, Figure 12) was also performed; **66** is composed of three thiophene units linked directly with single bond. Postigo et al. reported its activity against *Candida* spp. **66** produced significant decrease in *Candida* isolates from oropharyngeal candidiasis development [259]. In the swab, *C. albicans*, *C. tropicalis*, *C. parapsilosis,* and *C. krusei* were noticed [259].

New classes of compounds as candidates for PSs in photodynamic therapy are being searched. Taraszkiewicz et al. reported high PACT activity of imidazoacridinones (**67**, Figure 12) [260]. They showed reduction in *C. albicans* growth even higher than 4 log. Moreover, it was recognized that studied compounds are low efficient singlet oxygen generators (Φ_∆_ ca. 0.1), and they are able to form superoxide anion. It was observed that at the light dose of 20 J/cm^2^, the best results were provided. Studied compounds turned out not to be a substrate for ABC efflux pumps, considered as the crucial factor in forming resistance by this species. Additionally, their entry into the cells mainly by free diffusion was noted [260].

Interestingly, the PACT method may be applied in non-medical use. De Menezes et al. evaluated the potential of coumarins and furocoumarins (psoralens) as fungicides for agricultural purposes [261]. High potential was reported in photodynamic inactivation (up to 4 log of growth reduction) of *Colletotrichum acutatum* and *Aspergillus nidulans* by irradiated PSs: 8-methoxypsolaren (**68**), isopimpinellin (**69**), 7-methoxycoumarin (**70**), and citropten (**71**). Studied PSs efficiently destroyed active fungal cells as well as conidia [261,262]. The activity of porphyrazines and phthalocyanines is summarized in Table 7.

## 10. Conclusions

Recently, we provided comprehensive overviews of PACT usability in combating bacteria and viruses [18,44,237]. All PSs classes such as phenothiazines, xanthenes, curcumin, porphyrinoids (porphyrins, chlorins, and phthalocyanines) fullerenes, and many others were described. The review presented here is a continuation of the deep exploration of PACT knowledge. We had indicated that in the case of bacteria and viruses, the essential issue is the use of unstandardized light sources that can generate problems in providing repeatability and reproducibility of applied protocols. The same problem is observed for the use of the PACT method against fungi. On the other hand, scientists should focus their research on the development of a simple protocol enabling prediction of clinical results based on in vitro studies. This could simplify the search for the new PSs or the functionalization of already known ones. For proper selection of a new active PS, the structure–activity relationship should be considered. For example, as it was described, the dicationic porphyrin induced over 6 log reduction in planktonic *C*. *albicans*, whereas the effectiveness of the tetracationic porphyrin derivative needed a concentration 50 times higher to achieve the same effect. A very interesting phenomenon needing explanation is the key factor in the photodynamic method: a single oxygen sometimes works efficiently in the trace quantity. For example, photodynamically most active conjugates (linear PEI and HMW) were the worst ^1^O_2_ producers. We have reported the same observation for cancer cells and viruses lately [263]. Despite the drawbacks and limitations of PACT, it seems to be the perfect adjuvant method in the treatment of superficial infections caused by fungi. Such kind of disease possesses persistent character and needs a long-lasting treatment with an antifungal, which may cause serious side-effects. Therefore, PACT used as an adjuvant treatment is an ideal option.

**Figure 13:**
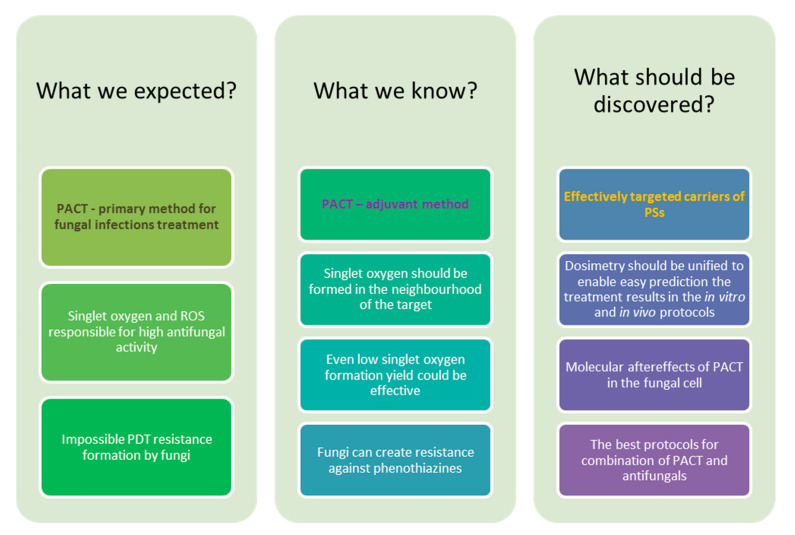
Conclusions in brief.

Moreover, use of PACT in the combination with conventional antifungals makes older drugs again active and effective. The possibilities mentioned are becoming more and more attractive from the perspective that we are living in the so-called “post-antibiotic” era, which involves fungal infection treatment as well. In Figure 13, the conclusions of this review are shown in brief.

## Figures and Tables

**Table 1:** Phenothiazines—photodynamic antimicrobial activity and dosimetry.

Reference	Photosensitizer	Incubation Time	Irradiation Wavelength (nm)	Light Dose (J/cm^2^)	Antimicrobial Activity Against	Reported Fungal Growth Inhibition
[28]	**1**	1 min	660	245	*C. albicans*	No decrease in number of CFUs; proteinase activity lowered
[35]	**1**	30 min	660	6; 15	*C. albicans* (in *G. mellonella*)	1 PACT significantly increased the survival rate
[36]	**1**, **2** and **5**	-	570–670	15	*F. keratoplasticum*, *F. moniliforme*	**1**, **2** and **5** in *G. mellonella*-infected larvae reduced the survival of *F.k.* microconidia to 38, 57, and 2%, respectively. The survival levels of *F.m.* microconidia in infected larvae were 69, 37, and 21%, respectively.
[45]	**1**, **9**	-	660; 532	21.42	*C. albicans*	max ca. 6 log red. (RB)* max ca. 6 log red. (PD) * *estimated data Gene expression studies
[46]	**1**	10 min	660	10; 20; 30	*C. albicans*	max. 53% red. (plankton) max. 74% red. (biofilm)
[47]	**1**	10 min	662	129.6; 162; 194.4	*C. albicans*	Complete eradication (lag phase cells) max. 2 log red. (stationary phase cells)
[48]	**1**	10 min	660	9; 27	*C. albicans*	In vivo experiment (clinical trial) Slightly increased sensitivity to fluconazole in in vitro tests
[49]	**1**	1–20 min	640	4.68	*C. albicans*	max ca. 1.5 log red.* *estimated data
[50]	**1** and **10**	30 min	600–650 (**1**); 490–540 (**10**)	10 to 60	*C. albicans*; *T. mentagrophytes*	*C.a.*/**1** max. >95% of inhibition *C.a.*/**10** max. >98% of inhibition *T.m.*/**10** max. > 98% of inhibition
[51]	**1**	1 h	660	15; 23; 57	*C. albicans*	max. 40.28% red. (biofilm)
[52]	**1**	5 min	660	26.3	*C. albicans*	0.49 log red. (serotype A) 2.34 log red. (serotype B)
[53]	**3**, **3** with verapamil or sodium azide	5 min	684 (1), 660 (3)	28	*C. albicans*	increasing calcium levels decreased the **3**-mediated PACT efficiency; Verapamil decreased **1**-mediated PACT effectiveness.
[54]	**1** (with glucose)	30 min	660	23; 46; 69; 92; 115; 138	*C. albicans*	max ca. 85% red. (biofilm)
[55]	**1** (with glucose)	10 min	660	10; 30; 60	*C. albicans*	max. 6-log reduction
[56]	**1** (with chitosan)	3 min	660	-	*C. albicans*	In vivo experiment (mouse model)
[57]	**1** (with CTAC, HPS, SDS, and Triton X-100 as surfactant)	1 h	-	-	*C. albicans*	max. ca. 47% red. PDT max. 100% red. SDS + PDT max. 100% red. CTAC + PDT max. 100% red. HPS + PDT max. ca. 39% Triton + X−100
[58]	**1**, **3**	30 min	Visible light; 675 (laser light)	20 (laser light)	*M. anisopliae*, *A. nidulans,*	Significant decrease of germination of conidia.
[59]	**3**	5 min	630	21.7	*C. albicans*	max. 50% of inhibition (biofilm)
[60]	**3**	5 min	630	20; 30; 40	*C. krusei*	max. 70% growth inhibition (plankton) max. 90% growth inhibition (biofilm)
[61]	**3**	60 s	635	24;18; 12	*C. albicans*, *C. glabrata*, *C. krusei*	*C.a.* max. 100% red. *C. g.* max. 100% red. *C. k.* max. 100% red.
[62]	**3**	1 min	635	200	*T. rubrum*	no fungal growth observed after PACT in vitro, and in an onychomycosis model Patient cured of onychomycosis confirmed in up to 6 months follow-up
[63]	**3**	5 min	630	18; 48;72	*T. rubrum*	>98% inhibition of fungal growth
[64]	**3** (with posaconazole or fluconazole)	30 min	630	50	*C. albicans*	3-log reduction
[65]	**1** (with fluconazole)	30 min	660	1.8; 3.6; 5.4; 7.2; 10.8; 14.4; 18	*C. albicans* (in *G. mellonella*)	**1** PACT alone and combined with fluconazole prolonged the larvae survival
[66]	**1** (fluconazole pre-treatment)	30 min-with PS alone; 2 h-with fluconazole	660	30; 60; 120	*C. albicans*	complete eradication
[67]	**1** (in combination with itraconazole, voriconazole, posaconazole, amphotericin B)	2 h	635	12; 24	*Fusarium* spp., *Exophiala* spp.	Reductions of up to 3.8 log and 6.4 log against planktonic *Exophiala* spp. and *Fusarium* spp Reductions of up to 4.2 log and 5.6 log against biofilms formed by *Exophiala* spp. and *Fusarium* spp.
[68]	**1**	10 min	660	28	*Candida* spp. (*C. albicans*, *C. tropicalis*, *C. glabrata*)*-*(*clinical trial*)	Clinical trial; PACT with **1** as effective as nystatin therapy
[69]	**1**	-	660	-	*C. albicans*	max. 73% red.
[70]	**1**	1 h	635	12, 24	*C. auris*	eradication of planktonic forms, ca. 2,7 log red. of biofilms, *C*.*a*. AR385 biofilm > 7 log red.
[71]	**1**	3 min	670	-	*Trichophyton* sp., *T. rubrum*, *T. mentagrophytes*, *C. parapsilosis*, *C. famata*, *A. fumigatus*, *Rhodotula*, *Microsporum*, *Microsporum + Epidermophyton, Fusarium (clinical trial)*	Clinical trial; 70% of patients cured
[72]	**1**	10 min	660	28	*Candida* ssp. (*clinical trial*)	Clinical trial
[73]	**1**	5 min	808	-	*C. albicans* (*Clinical trial*)	Clinical trial; >6 log red.
[74]	**2** and **58**	5 min (**58**); 30 min (**2**)	810 (**58**); 630 (**2**)	52 (**58**); 5 (**2**)	*T. rubrum*	0.64 log red. (**58**) 0.4 log red. (**2**)
[75]	**1**, **2**, **3** and **5**	30 min	400–700 (max. 631)	3; 6; 11	*N. dimidiatum*, *N. dimidiatum var. hyalinum*	max. 4.5 log red. (**1**/**3**; *Neoscytalidium dimidiatum*) max. 5 log red. (**1**/**3**; *Neoscytalidium dimidiatum var. Hyalinum*) max. 4–5 log red. (**2**; *Neoscytalidium dimidiatum*) max. 5 log red. (**2**; *Neoscytalidium dimidiatum var. Hyalinum*) max. 5 log red. (**5**; *Neoscytalidium dimidiatum*) max. 3.5 log red. (**5**; *Neoscytalidium dimidiatum var. Hyalinum*)
[76]	**1**	2 h	635	12	*Scedosporium* and *Lomentospora* spp.	3.3 log red. *(L. prolificans*) 2.8 log red. (*S. boydii*) 3 log red. (*S. aurantiacum*) 3 log red. (*S. dehoogii*) 5.2 log red. (*S. apiospermum*) 2.7 log red. (*S. minutispora*)
[77]	**1**	10 min	625	3; 12; 24; 40; 60	*T. rubrum*	up to 100%
[78]	1@AuNPs	-	650; 530	80; 100	*T. mentagrophytes*	In vivo experiment (rabbit model)
[79]	1, **58** in compilation with nystatin and chlorhexidine)	-	660; 808	10	*C. albicans*	1.04 log red. (**1**) 1.2 log red. (**ICG**)
[80]	**1**, **3**, **2** and **5**	30 min	635	0; 10; 15; 20	*F. oxysporum*, *F. moniliforme*, and *F. solani*	*F. oxysporium* (MIC): **1** 12.5–25 µM **3** 25–50 µM **2** 2.5 µM 5 5 µM *F. moniliforme* (MIC): **1** 25 µM **3** 10–12.5 µM **2** 10 µM **5** 5 µM *F. solani* (MIC): **1** 25–75 µM **3** 12.5–25 µM **2** 2.5–5 µM **5** 5 µM
[81]	**1**, **2** (combined or not with potassium iodide)	10 min	660	3.18; 6.36; 12.73; 19.10 in vitro 105.26; 157.89 in vivo	*Candida albicans.*	In vitro (*C.a.*biofilm): **1** + KI 2.31 log red. **2** 1.77 log red. In vivo, almost complete eradication of *C*.*a*.
[82]	**1**	24 h	576–672	15	*C. albicans*, *C. parapsilosis*	*C. albicans* biofilm MIC 30 µM *C. parapsilosis* biofilm MIC up to 7.5 µM
[83]	AuNPs@1; AuNPs@3	30 min	662 (1); 635 (3)	21.6	*C. albicans*	max. 80% red. in biofilm formation * * Estimated data in case of both types of nanoparticles used
[84]	**7** and **8**	3 or 18 h	600	12; 36	*C. albicans biofilms*	up to total cell inactivation with FSc
[85]	**2**, **58** (EmunDo^®^)	30 min	810; 630	15 (630 nm); 55 (810 nm)	*C. albicans*	max. 1.9 log (**58**) max. 3.37 log red. (**2**)
[86]	**1**, **2**, **3**, **5**	30 min	400–790	5; 10; 15; 20; 25; 30	*C. acutatum*, *C. gloeosporioides*	max. antifungal activity ca. 5 log red. (**2** and **5** against conidia)
[87]	**1**	10 min	660	18 (one session); 36 (two sessions)	*C. albicans*	In vivo experiment (mouse model)
[88]	Crystal violet, New Fuchsin; Azure B	30 min	455–800	-	*C. albicans*	max. ca. 5 log red. (CV) max. ca. 2.7 log red. (AzB) max. ca. 1 log red. (NF)
[89]	**1**	-	630	18	*various species of fungimainly T. rubrum,* and *T. mentagrophytes* (*Clinical trial*)	In vivo experiment (clinical trial)
[90]	**1**, **2**, **3**, **5**	30 min	634; 631	5; 10; 15; 25	*C. albicans*, *C. glabrata*, *C. krusei*, *C. parapsilosis*, *C. tropicalis*	MICs up to 1 µM
[91]	**3**	10 min	630	21.47	*C. albicans*	max. ca. 62% of inhibition (biofilm formation)
[92]	**3**	5 min	637	18	*C. albicans*	0.46 log
[93]	**6**	0, 15, 30, 60 min, 3, 5 and 24 h	639.8	18; 37	*C. albicans*	max. 6 log reduction
[94]	**3** (with chitosan)	10, 30 or 60 min	630	50	*C. albicans*	Complete eradication (ca. 7 log10)
[95]	**1**	1 min	660	7.5	*Candida* spp. (*clinical trial*)	Clinical trial
[96]	**1**@AuNP	12 and 24 h	660	38.2	*C. albicans*	**1**: 63.2% (crystal violet assay) or 81.9% (XTT assay) biofilm inhibition **1**@AuNP: 82.2% (crystal violet) or 95.4% (XTT assay) biofilm inhibition
[97]	**1**	1 h	-	-	*F. pedrosoi*, *C. carrionii*	*Ca*. 4 log red.
[98]	**1**, **2**, **3** and **5**	30 min	634/631	5; 10; 15; 20; 25; 30	*T. mentagrophytes*, *T. rubrum*	*T*.*m*. MIC: **1** up to 5 µM **3** up to 2.5 µM **2** up to 2.5 µM **5** up to 0.1 µM *T*.*r*. MIC: **1** up to 5 µM **3** up to 1 µM **2** up to 2.5 µM **5** up to 0.5 µM
[99]	**1**	5 min	660	60; 120; 180	*C. albicans*, *C. tropicalis, C. krusei*, *C. guillermondii*	up to 78% reduction
[100]	**1** (ointment)	4 h	660	28	*Fungi causing chromoblastomycosis* (*Clinical trial*)	80–90% volume reduction of the lesion in 10 patients
[101]	**1** (with verapamil)	30 min	660	60	*C. albicans*	ABC pump decreased the effectiveness of **1** more than MFS pump
[102]	**3**, **1**, and **2**	30 min	635; 660	in vitro: 0; 1.95; 3.90; 5.85; 7.80; 9.75 in vivo: 78; 120	*C. albicans*	in vitro: **2** 4.43 log red **1**, **3** < 1 log red
[103]	**3**	5 min	630	18–90	*T. rubrum*	max. 1.72 log red.
[104]	**9**, **10**	5 min	455	95	*C. albicans*	**9** max. 3.45 log red. (plankton) **10** max. 1.97 log red. (plankton) biofilms < 1 log red.
[105]	**1**, **3**, malachite green	5 min	660	15.8; 26.3; 39.5	*C. albicans*	**1** max. 2.71 log red. **3** max. 3.07 log red. malachite green max. 2.25 log red.
[106]	**1**	5 min	684	28	*C. albicans*	max. ca. 2.5 log red
[107]	**3**	5 min	630	36–180	*Candida* spp. *(Clinical isolates)*	max. 3.54 log red. single irradiation; complete eradication after second irradiation 55% inhibition of adhesion to buccal cells
[108]	**1**	5 min	683	28	*C. albicans*	<10% cel viability (estimated data)
[109]	**1**	-	685	28	*C. albicans*, *C. dubliniensis*, *C. krusei,* and *C. tropicalis*	*C.a.* 88.6% CFU number reduction *C*.*d*. 84.8% CFU number reduction *C*.*k*. 91.6% CFU number reduction *C*.*t*. 82.3% CFU number reduction

**Table 2:** Xanthenes—photodynamic antimicrobial activity and dosimetry.

Reference	Photosensitizer	Incubation Time (Min.)	Irradiation Wavelength (nm)	Light Dose (J/cm^2^)	Antimicrobial Activity	Reported Inhibition in Bacterial Growth
[130]	**9**	5	532	42.63	*C. albicans*, *C. dubliniensis*	Complete eradication for both (plankton) Biofilm: *C.a.* 0.74 log red. *C.d*. 0.21 log red.
[132]	**9**	10	532	42.63	*C. glabrata*	One application: 4.64 log red. Four applications: 5.94 log red.
[133]	**9**	1	532	14.34	*C. albicans*	In vivo experiment (mouse model)
[134]	**9**	0.5 or 1	450	28.8	*C. albicans*	In vitro: Complete eradication for plankton and biofilm In vivo: No significant red.
[136]	**9**	5	532	42.63	*C. albicans*, *S. sanguinis*	1.07 log red. (*C.a.* biofilm) 0.39 log red. (*C.a*. and *S.s.* mixed biofilm)
[137]	**9**	5	532	42.63	*C. albicans*, *C. glabrata, C. tropicalis*, *C. dubliniensis*	*C.a.* 4.62/1.01 log red.* *C.g*. 2.58/1.17 log red.* *C.t.* 2.81/1.17 log red.* *C.d*. 3.99/1.13 log red.** (plankton/biofilm)
[140]	**10** and **12**	5	532	42.63	*C. albicans*	0.22 log red. (**10**) 0.45 log red. (**12**)
[141]	**10**	30	532	68; 133; 228	*T. rubrum*	max. ca. 85% red.
[142]	**10** and **11**	-	518 (RB); 375 (riboflavin)	5.4	*F. solani*, *A. fumigatus*, *C. albicans*	C.a. 95.6% growth inhibition A.f. 79.8% growth inhibition F.s. 78.2% growth inhibition
[143]	**10** with gold nanoparticles	120	500–780	3.18; 15.9; 31.8; 63.6; 95.4	*C. albicans*	max. 4.89 log red. (plankton) max. 1.53 log red. (biofilm)
[144]	**10** with clotrimazolum	30	530	4; 8; 12; 24	*T. rubrum*	max. 100% inhibition

**Table 3:** Curcumin—photodynamic antimicrobial activity and dosimetry.

Reference	Photosensitizer-13 Concentration	Incubation Time (Min.)	Irradiation Wavelength (nm)	Light Dose (J/cm^2^)	Antimicrobial Activity Against	Reported Inhibition in Bacterial Growth
[149]	20 µM, 30 µM and 40 µM	20	-	5.28 and 18	*C. albicans*, *C. tropicalis*, and *C. glabrata*	40 µM of CUR at 18 J/cm^2^ reduced the metabolic activity of *C. albicans* biofilm (by 85%), *C. glabrata* biofilm (by 85%), and *C. tropicalis* biofilm (by 73%).
[150]	20 µM, 30 µM and 40 µM	20	455	5.28	*C. dubliniensis*	The combination of Cur and LED light led to a reduction in CFU values of fungal planktonic cultures from 10^6^ to 10^3^ (CD6 strain) and 10^2^ (CD7 and CD8 strains). The mean percentage reduction in biofilm cultures ranged from 57.70 to 82.05%
[151]	1 µM, 5 µM, 10 µM, 20 µM, 40 µM and 80 µM Also in combination of 208 µM fluconazole	20	430	9	*C. albicans*	PDT utilizing 1 μM curcumin and 9 J/cm^2^ of light decreased the number of planktonic *C. albicans* colonies by three orders of magnitude and at curcumin concentrations of 5 μM or higher, PDT eliminated all *C. albicans* colonies. Combined treatment of fluconazole with PDT and curcumin led to decrease of *C. albicans* cell viability to 5%. albicans in adherent culture.
[152]	60 µM	20	455	2.64, 5.28, 7.92, 10.56, 13.2	*C. albicans*	The inhibition rates after CUR-PDT in three biofilms (ATCC 90028, CCA1, and CCA2) were 90.87%, 66.44%, and 86.74%, respectively (*p* < 0.05).
[153]	40 µM and 80 µM	20	450	37.5 or 50	*C. albicans*	CUR-mediated aPDT promoted a downregulation of gene expression of fungal genes related to adhesion and biofilm formation (ALS1 and HWP1) and the genes responsible for the oxidative stress response (CAP1, CAT1, and SOD1).
[154]	5, 10, 20, 30 and 40 µM	1, 5, 10 and 20	455	5.28	*C. albicans*, *C. glabrata*, and *C. dubliniensis*	For the planktonic cultures, photoinactivation depended on Cur concentration and complete inactivation of the suspensions was achieved. Cur-mediated PDT was effective against Candida biofilms, with reductions of 94%, 89%, and 85% in the cell viabilities of *C. albicans*, *C. glabrata,* and *C. dubliniensis*, respectively. The highest decrease in cell viability for the biofilms was observed for the combination of 40 µM Cur with 20 min of PIT.
[155]	0.1 μg/mL; 1 μg/mL; 10 μg/mL curcumin and curcumin incorporated in nanoparticles	10	417	10, 20, and 40	*Trichophyton rubrum*	Antimicrobial photodynamic inhibition utilizing curcumin (10 μg/mL) with 10 J/cm^2^ of blue light completely inhibited fungal growth via induction of ROS and nitrogen species (RNS). Curcumin in nanoparticles induced greater NO• expression and increased apoptosis of fungal cells.
[156]	2.5 µM	20	440–460	37.5	*C. albicans*	The DNA damage was analyzed by the comet assay. DNA damage was significantly increased for longer comet tails observed after application of Cur mediated PDT.
[157]	1.5 g/L	-	450	20.1	*C. albicans*	PDT reduced 1.46 *C. albicans* log_10_ CFU/mL, in contrast to 450 nm blue LED or curcumin for 5 min.
[158]	0.005, 0.01, 0.05, 0.1, 0.5, 1, 5, 10, and 20 µM for planktonic culture 5, 10, 20, 30 and 40 µM for biofilms	20 (planktonic) 5 or 20 (biofilm)	455	5.28 and 37.5	*C. albicans*	In the conditions of complete inactivation of the fungal planktonic form (20 µM/20 min preirradiation time/5.28 J cm^−2^), the metabolic activity of biofilms was reduced by 70%.
[159]	20, 40, and 80 µM	20	455	37.5	*C. albicans*	All curcumin and LED light treatments resulted in a significant reduction of *C. albicans* viability after PDT. The curcumin concentration of 80 µM combined with LED light application promoted the highest log_10_ reduction in colony counts (over 4 logs).
[160]	80, 100, and 120 μM	20	from 440 to 460	37.5	*C. albicans*, *C. glabrata*, and *Streptococcus mutans*	For the biofilms of both ages (24 and 48 h), the tested concentrations of Cur with light promoted a reduction in cell metabolism, with the highest reduction 40.62% achieved with the concentration of 120 μM of Cur.
[161]	40 μM	20	455	5.28	*C. albicans* co-cultured with human keratinocytes	After applying aPDI the reduction in CFU/mL equivalent to 1.7 log_10_ was observed.
[162]	260 μM Free curcumin and curcumin encapsulated in nanoparticles NP	20	455	37.5	*C. albicans*	aPDT with free CUR topically applied on the tongue of mice with oral candidiasis led to a reduction of 1.11 log_10_ in the viability of fungus, while aPDT mediated by anionic CUR-NP demonstrated no antifungal effect, and cationic CUR-NP reduced the fungal cells (0.44–0.74 log_10_) even in the absence of light.

**Table 4:** Porphyrins—photodynamic antimicrobial activity and dosimetry.

Reference	Photosensitizer	Incubation Time	Irradiation Wavelength (nm)	Light Dose (J/cm^2^)	Antimicrobial Activity Against	Reported Fungal Growth Inhibition
[121]	Photogem^®^	30 min	455 (440–460)	10.5, 18.0, 25.5, 37.5	*C. albicans*, *C. glabrata*	plaktonic culture—up to complete eradication; biofilms < 1.0 log red.
[163]	Photofrin	30 min	n.d. (white light)	9	*C. albicans*, *C. krusei C. glabrata*	significant reductions of *C. albicans* at 1 µg/mL, *C. krusei* at 3 µg/mL, *C. glabrata* at 10 µg/mL
[164]	Photofrin	1 or 5 min	400–700	9	*C. albicans* (germ tubes and biofilm)	optimization of PDI conditions using Photofrin; significant reduction of germ tubes; higher activity towards biofilm than amphotericin B
[165]	Photofrin	1 or 24 h	630	45–135	*C. albicans*, *C. glabrata*, *C. parapsilosis*, *C. Krusei*, *C. tropicalis*	Optimization of conditions, up to eradication of fungi apart from *C*. *krusei* which was reduced by up to 90%. Amphotericin B and azole-resistant strains were equally susceptible to PDI.
[166]	Photogem^®^	30 min	455	18.0, 25.5, 37.5	*C. albicans*, *C. dubliniensis*, *C. tropicalis*, *C. krusei*	Complete eradication of *C. albicans*, *C. dubliniensis*, and *C. tropicalis*; *C. krusei* could not be eradicated
[167]	Photogem^®^	30 min	455 (440–460)	37.5	ex vivo cultures on dentures: *C. albicans*, *C. glabrata*, *C. tropicalis*, *C. dubliniensis*, *C. krusei*	up to 3.99 log red.
[168]	Photogem^®^	30 min	455 or 630	305	*C. albicans* in vivo oral candidiasis in mice	1–1.4 log red.
[169]	Photogem^®^	30 min	455 (440–460)	122	case reports of denture stomatitis: *C. Albicans*, *C. Glabrata*, *C. tropicalis*	two patients cured after the applied PDI
[170]	Photogem^®^	30 min	455 (440–460)	37.5 122	denture stomatitis: *C. albicans*, *C. tropicalis*, *C. Glabrata*, *C. Lusitaniae*, *C. Rugosa*, *C. guillermondii*, *Cryptococcus humicola*, *Cryptococcus albidus Kloeckera apis/apiculata*	clinical study, 45% success rate
[171]	Photogem^®^	1 h	630	54	onychomycosis	case report; complete recovery of one patient
[172]	Photogem^®^	0 5 10 20	410–440 (incubation) 610–640 (PDI)	4 8 17	*C. albicans*	<1 log red. when incubation in the dark 7 log red. when incubation with low intensity light (4 J/cm^2^)
[173]	**15**	30 min	400–780	72	*C. albicans*	7 log red.
[174]	**15**	30 min	400–800	72	*C. albicans* biofilm	up to 3.26 log red.
[178]	5-ALA	4 h	broadband red light	75	case report of interdigital mycoses treatment (*T. mentagrophytes*, *C. albicans*, *T. rubrum*)	after 4 weeks follow-up only two out of 9 patients were recovered
[179]	ALA, itraconazole	4 h	630	360	*C. albicans* (cutaneous granuloma)	case report—full recovery of one treated patient
[180]	methyl 5-aminolevulinate	3 h	630	37	*Malassezia*	six case studies; improvement observed in five patients
[181]	methyl 5-aminolevulinate	3–5 h	630	37	case report of *Acremonium sclerotigenum* onychomycosis	clinical cure in 12-month follow-up
[182]	methyl 5-aminolevulinate and bifonazole	3 h	635	37	*Neoscytalidium dimidiatum* onychomycosis	case report; reinfection with the pathogen without the clinical symptoms
[183]	ALA	n.d.	635	10	*Fonsecaea monophora*	estimated 4 log red.
[184]	ALA combined with terbinafine, itraconazole, or voriconazole	n.d.	635	n.d.	chromoblastomycosis: *F*. *nubica*, *F*. *pedrosoi*, *F*. *monophora*	case report, full recovery of three out of five patients
[185]	ALA	12 h	635	30 60 90	*Fonsecaea monophora* conidia	up to almost 6 log red.
[186]	ALA	5 h	635	50 100 200 300	*C. albicans* biofilm	up to 75% cell viability decrease
[187]	**1**, **16**	10 min	630 (**16**) 660 (**1**)	85 (**16**) 6048 (**1**)	in vivo vaginal candidiasis (*C. albicans*) in mice	up to 1 log red. **16** used in 10-fold lower concentration to yield the same effect
[188]	5,10,15,20-tetrakis (*N*-methylpyrid−4-yl) porphyrin tetra (4-toluenesulfonate), Photofrin	10 min	75% in range of 575–700	n.d.	*C. albicans*, *C. glabrata*	up to 3 log red; increased susceptibility to PDI when azole-resistance expressed
[189]	**17**	10 min	67% in range 575–700	2.4	*C. albicans*, *C. glabrata S. cerevisiae*	mechanistic studies
[190]	**17**	30 min	350–800	n.d.	*C. albicans*	5 log red.
[191]	**17**, **18**	-	-	-	*T. rubrum* *Scopulariopsis brevicaulis*	localization and stability study
[192]	**17**, **18**, Eosin Y	30 min	white light	21.6 43.2 64.8 1382.4	*T. rubrum Trichophyton interdigitale S*. *brevicaulis*	up to complete eradication by each of the tested PSs
[193]	**19**, **20**, **21**	30 min	n.d. (visible light)	n.d.	*C. albicans*	4 log red. for **19** and **20**, almost no effect at the same concentrations of **21**
[194]	**17**, **19**, **20**	30 min	350–800	n.d.	*C. albicans*	mechanistic study, up to 5 log red.
[195]	**17**, **19**, **20**	30 min	350–800	162	*C. albicans*	3.5 log red.
[196]	**17**, miconazole	30 min	350–800	58.5	biofilms: *C*. *albicans*, *C*. *glabrata*, *C*. *tropicalis*, *C*. *parapsilosis*	*C. glabrata*—resistant to PDI Other strains cell viability reduced to 64%. All strains susceptible to combined PDI and miconazole therapy (additive or synergistic)
[197]	**17**	n.d.	67% in range 575–700	1.0	*C. albicans*	>1.5 log red. of PDI > 2.0 log red. when PDI combined with miconazole therapy
[198]	5,10,15,20-tetrakis [4-(3-*N*,*N*-dimethylaminopropoxy) phenyl]-porphyrin; 5,10,15,20-tetrakis [4-(3-*N*,*N*,*N*-trimethylammoniumpropoxy) phenyl] porphyrin tetraiodide	30 min	350–800	162	*C. albicans*	up to 4.8 log red.
[199]	**17**; 5,10,15,20-tetrakis (*N*-pentylpyrid−4-yl) porphyrin tetraiodide	3 h	400–800	240	*Penicillium chrysogenum* conidia	**17**: 4.1 log red 5,10,15,20-tetrakis (*N*-pentylpyrid−4-yl) porphyrin tetraiodide: 3.4 log red.
[200]	**17**	30 min	67% in range 575–700 (in vitro) 514 (in vivo)	2.4 (in vitro) 90 (in vivo)	*C. albicans* (in vitro and in vivo in mice)	in vitro: nearly 4 log red. in vivo: 50-fold reduction of CFUs
[201]	5-phenyl−10,15,20-tris (*N*-methyl−4-pyridyl) porphyrin chloride	1, 15, 30	n.d. (white light)	0–9	*C. albicans*	mechanistic study; up to 6 log reduction
[202]	**17**, **22**	15 min (plankton) 4 h (biofilm)	418	12.1 (plakton) 48.2 (biofilm)	*C. albicans*	planktonic: up to 6 log red biofilm: up to 5 log red.
[203]	5-(*N*-methylpyrid−4-yl)−10,15,20-triphenylporphyrin iodide 5,10-bis (*N*-methylpyrid−4-yl)−15,20-diphenylporphyrin diiodide 5,15-bis (*N*-methylpyrid−4-yl)−10,20-diphenylporphyrin diiodide 5,10,15-tris (*N*-methylpyrid−4-yl)−20-phenylporphyrin triiodide 5,10,15,20- tetrakis (*N*-methylpyrid−4-yl) porphyrin tetraiodide	n.d.	400–800	30 60 90 120	*Colletotrichum graminicola*	up to over 4.5 log red.—eradication
[204]	deuteroporphyrin monomethyl ester (DP mme), 23, deuteroporphyrin (DP), hematoporphyrin (HP), Photofrin, zinc(II) phthalocyanine (ZnPc), phthalocyanine tetrasulfonate (PcS_4_), aluminum(III) phthalocyanine chloride tetrasulfonate (AlPcS_4_).	30 min at 28 °C	n.d.	108	*T. rubrum*	DP mme, Sylsens B: fungicidal effect at 3 µg/mL Photofrin, ZnPc, PcS_4_, AlPcS_4_: delay the growth, no difference from the control after 7 days of culture
[205]	**23**, DP mme	2 h	580–600	108	*T. rubrum* in ex vivo skin model	up to 100% decrease of cell viability
[206]	**23**, DP mme	2 or 3 h	580–600	108	*T. rubrum*	PDI conditions optimization; up to complete eradication
[207]	**23**	2	340–550	18	*T. rubrum*	Optimization of the PDI conditions, up to complete eradication at 1 µM
[208]	5,10,15-tris(*N*-methylpyrid−4-yl)−20-(4-sulfanylphenyl)porphyrin trichloride, 23, 25	1 h	532	0.15 0.6	*Trichophyton mentagrophytus* conidia	5,10,15-tris(*N*-methylpyrid−4-yl)−20-(4-sulfanylphenyl) porphyrin trichloride: 4.6 log red. **23**: 4.4 log red. **25**: up to 4.1 log red.
[209]	**25**	1 h		27 28 81	Ex vivo onychomycosis: *T. rubrum*, *T. mentagrophytes*, *T. tonsurans*	**25**: single irradiation—≥70% cell viability decrease, second irradiation—eradication
[210]	**26** in poly (silesesquioxane) thin films	n.d.	350–800	n.d.	*C. albicans*	2.5 log red. as compared to 0.5 log red for free **26**
[211]	CdTe quantum dots, Zn (II) 5,10,15,20-tetrakis (*N*-ethylpyrid−2-yl)porphyrin and their combination	10 min	460	0–81	*C. albicans*	combination 1 log red. free porphyrin 3 log red.
[212]	silver core-silica shell nanoparticles, hematoporphyrin IX, material based on both	30 min	400	n.d.	*T. rubrum*	nanoparticles alone < 1 log red. hematoporphyrin IX alone < 1 log red. material 3 log red.
[213]	methyl 5-aminolevulinate	3 h	630	37	*T. rubrum* (clinical case of onychomycosis)	three sessions of irradiation resulted in clinical cure at 24-month follow-up

**Table 5:** Chlorins—photodynamic antimicrobial activity and dosimetry.

Reference	Photosensitizer	Incubation Time (Min.)	Irradiation Wavelength (nm)	Light Dose (J/cm^2^)	Antimicrobial Activity Against	Reported Inhibition in Bacterial Growth
[215]	**28**	-	660	10; 20; 30	*F. solani*, *A. fumigatus*	F.s. max. Complete eradication A.f. max. ca. 50% red.
[216]	**27**	20	660	40	*C. albicans*	max. ca. 90% red. for plankton and biofilm
[217]	**29**	15	627	31.5	*C. albicans*, *E. faecalis*	58.98% red. (*C.a*. and *E.f.* mixed biofilm)
[218]	**27** (encapsulated in CTAB-liposomes)	15	662	50	*C. albicans*, *C. krusei*, *C. tropicals*,	In vitro: Max. Complete eradication In vivo: max. 1.75 log red.
[219]	PEI−**27** (linear, LMW, HMW)	10	665	0–100	*C. albicans*, *S. aureus*, *S. pyogenes*, *E. coli*, *P. aeruginosa*	*C. albicans*: **27** 2 log red. **27**-LMW 2 log red. **27**-HMW; **27**-lin 4–6 log red.
[221]	**34**–**37**	30	415; 660	10; 20	*C. albicans*, *MRSA*, *E. faecium*, *E. coli*	*C. albicans*: **34**–**36**—max. complete eradication 37 No significant red.
[222]	**38**	20	660	25	*C. albicans*	Plankton: max. Complete eradication Biofilm: PACT No significant red. PACT + SDT 3.39 log red.
[223]	**38** (pre-treatment with fluconazole)	20	660	50	*C. albicans*	2.6 log
[224]	**38**	20	660	37.5	*Candida* spp. (*clinical trial*)	max 1.96 log red. without formulation > 5 log red. for formulations
[225]	**38**	20	660	37.5	*C. albicans*, *C. glabrata*, and *S. mutans* multispecies biofilm	*C.a.* ca. 1 log red. *C*.*g*. ca. 1.5 log red. estimated data
[226]	**38**	20 (planktonic)	660	18.0; 25.5; 37.5	*C. albicans*, *C. glabrata*, and *C. tropicalis (clinical isolates)*	max. 0.9 log red. (*C.a.*) max. 1.4 log red. (*C.t.*) max. 1.5 log red. (*C.g.*)

**Table 6:** Porphyrazines and phthalocyanines—photodynamic antimicrobial activity and dosimetry.

Reference	Photosensitizer	Incubation Time	Irradiation Wavelength (nm)	Light Dose (J/cm^2^)	Antimicrobial Activity Against	Reported Inhibition in Bacterial Growth
[229]	phthalocyanine encapsulated chitosan/TPP nanoparticles (FNP) from chitosan, tripolyphosphate (TPP), and phthalocyanine-4,4′,4″,4‴-tetrasulfonic acid (FePC). In experiments combining PDT with chemical therapy, *C. tropicalis* was treated with 128 µM flucytosine prior to or after the PDT experiments	4 h	630	20	*C. tropicalis*	After PDT with FePC (70% cell viability) or FNP (50% cell viability) Prior to and after the flucytosine therapy, FNP-PDT largely decreased the viability of *C. tropicalis* cells. Cell viability was ~25% for the treatment with flucytosine and subsequent PDT with 80 μM FNP.
[230]	**39**, **40**	1.5 h	635	50	*C. albicans*	The fungal biofilm inactivation with 3 log (**39**) was observed only after fractionated LEDs irradiation. *C. albicans* in suspension was completely inactivated after **39** (1.8 mM) at soft light radiation.
[231]	**41**, **42**	15 min.	665	50	*C. albicans*, *Pseudomonas aeruginosa*	Sufficient efficacy of PACT for planktonic cultures, but low PACT efficacy (<3 logs) of the photoinactivation of the 48 h bacterial and fungal biofilms in comparison to effect in suspension.
[232]	**43** entrapped in cationic and anionic nanoemulsions (NE)	30 min.	660	50, 100	*C. albicans*	In case of planktonic cultures, cationic NE-**43** reduced significantly both colony counts and cell metabolism. In addition, cationic NE-**43** and free **34** caused significant damage to the cell membrane. In case of the biofilms, cationic NE-**43** reduced cell metabolism by 70%, whereas anionic NE-**43** was inactive.
[233]	**43**-NE	-	660	30.9	onychomycosis	Clinical cure of 60% of treated lesions
[234]	**43**-NE	30 min.	675	5, 10	*Cryptococcus neoformans*	Treatment with **43**-NE, using selected PS concentrations (e.g., 4.5 µM) and light doses (e.g., 10 J cm^−2^) resulted in a reduction of up to 6 logs in survival of fungi.
[235]	**43**-NE	30 min.	675	25, 50 and 100	*C. albicans* and *C. tropicalis*	APDT with **43**-NE led to a reduction of five orders of magnitude in viability for *C. albicans* and between four and five orders of magnitude for *C. tropicalis*.
[236]	**44**	at least 1 h	670–675	1–2	*C. albicans*	Application of 1.0 μM Pc 4 and irradiation at 2.0 J/cm^2^ caused cell survival reduction by 4 logs.
[237]	**43** encapsulated in cationic nanoemulsions (NE) or **43** dissolved in DMSO	-	660	100	*C. albicans*	**43**-NE-mediated PDT reduced 2.26 log 10 of C. albicans recovered from the oral lesions of immunocompromised mice when compared with the control group.
[238]	**45**	2.5, 15, and 30 min	350–800	54	*C. albicans*	After 15 min irradiation—a 5 log decrease in the cell viability (treated with 10 mM **45**). After 30 min irradiation—a 4 log decrease in the cell viability (treated with 1 mM **45**). After over 30 min irradiation—a 2.5 log decrease in the cell viability (cell suspension of 10^7^ cells mL^−1^ incubated with **45**)
[239]	**45**	-	350–800	54	*C. albicans*	After 30 min irradiation, 5 µM **45** produced ~5 log decrease in cell viability
[240]	**46**	5 min (pre-irradiation time)	660	26.3	*Candida* spp., *Trichosporon mucoides*, *Kodamaea ohmeri*	A mean reduction of 0.45 log in case of *Candida* spp. biofilms, and a reduction of 0.85 and 0.84 for biofilms formed by *T. mucoides* and *K. ohmeri*, respectively.
[241]	**44**	2–16 h	670–675	2	*Trichophyton rubrum*	Reduction of metabolic activity by 50% within 4 h compared to controls following irradiation of Pc 4-loaded terbinafine-sensitive (24602) and terbinafine-resistant (MRL666) microconidia or hyphae.
[243]	**49**, **50**	10 min.	675	12,30 and 60	*Staphylococcus aureus*, *Pseudomonas aeruginosa*, *C. albicans*	Complete inactivation of *S. aureus* and *C. albicans* by the cationic photosensitizer. *P. aeruginosa* inactivated with 4 log
[244]	Carboxymethyl chitosan-zinc (II) phthalocyanine conjugates	4 h	-	-	*C. albicans*	The highest photocytotoxicity (with an IC90 value down to 0.72 μM) observed for conjugate of ZnPcN and CMC1 (CMC1, MW = 50 kDa, a low molecular-weight CMC, N,O-carboxymethyl chitosan)
[245]	phthalocyanine derivatives	30 min.	-	30, 60, 90	*C. albicans*	ZnPc reduced the mean cell viability values of *C. albicans* to 5 (at 30 J/cm^2^ light doses) and 2 CFU/mL (at 60 J/cm^2^ light doses). **54** and **55** reduced the mean CFU/mL values to 5 CFU/mL after irradiation with 60 J/cm^2^. Studied compounds had inhibitory effects on fungus after irradiation and reduced the mean CFU/mL levels to ≤100 CFU/mL.
[246]	**57**	1 h	675	12	*C. albicans*	**57** inactivated *C. albicans* at sub-micromolar level. The IC_50_ value was 0.013 µmol^−1^ (for a cell density of 10^7^ cells mL^−1^)
[242]	**47**, **48**	3 h	>610 nm	27	*C. albicans*	High PACT of the dodeca-cationic phthalocyanine against *C. albicans* with an IC90 value down to 1.46 µM

**Table 7:** Other photosensitizers—photodynamic antimicrobial activity and dosimetry.

Reference	Photosensitizer	Incubation Time (Min.)	Irradiation Wavelength (nm)	Light Dose (J/cm^2^)	Antimicrobial Activity Against	Reported Inhibition in Bacterial Growth
[74]	**58** (EmunDo^®^)	5	800	5	*T. rubrum*	0.64
[247]	**58** (EmunDo^®^)	5	810	55	*C. albicans*	1.90
[252]	**60**	30	400–780	72	*C. albicans*	Dependently on strain up to 7.00
[254]	**61**	<1	602 ± 10	18	*C. albicans*, *C. parapsilosis*, *C. krusei*	ca. 3
[256]	**62**, **63**, **64**	30	532	24	*C. albicans cal-1*	3.76, 3.08, and 3.72, respectively
[258]	**65**	30	350–800	162	*C. albicans*	5.00
[260]	**67**	30	405	20	*C. albicans*	Dependently on derivative up to 5.00
